# Multifold Enhanced Photon Upconversion in a Composite Annihilator System Sensitized by Perovskite Nanocrystals

**DOI:** 10.1021/acsnano.4c03753

**Published:** 2024-05-31

**Authors:** Xian Wei Chua, Linjie Dai, Miguel Anaya, Hayden Salway, Edoardo Ruggeri, Pengqing Bi, Zhihong Yang, Samuel D. Stranks, Le Yang

**Affiliations:** † Cavendish Laboratory, Department of Physics, University of Cambridge, JJ Thomson Avenue, Cambridge CB3 0HE, U.K.; ‡ Institute of Materials Research and Engineering (IMRE), Agency for Science, Technology and Research (A*STAR), Innovis #08-03, Singapore 138634, Singapore; § Department of Chemical Engineering and Biotechnology, University of Cambridge, Philippa Fawcett Drive, Cambridge CB3 0AS, U.K.; ∥ Department of Materials Science and Engineering, National University of Singapore, 9 Engineering Drive 1, #03-09 EA, Singapore 117575, Singapore; ⊥ Departamento Física de la Materia Condensada, Instituto de Ciencia de Materiales de Sevilla, Universidad de Sevilla−CSIC, Calle Américo Vespucio 49, Sevilla 41012, Spain

**Keywords:** lead halide perovskites, triplet−triplet annihilation, upconversion, triplet sensitization, excitons

## Abstract

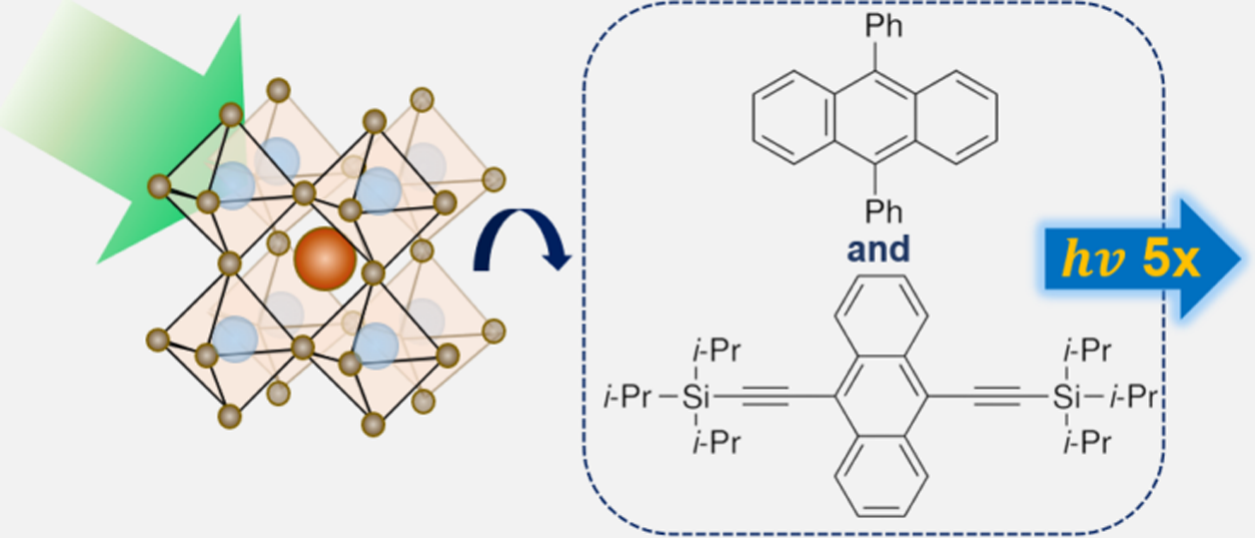

Photon upconversion via triplet–triplet annihilation (TTA-UC) provides a pathway to overcoming the thermodynamic efficiency limits in single-junction solar cells by allowing the harvesting of sub-bandgap photons. Here, we use mixed halide perovskite nanocrystals (CsPbX_3_, X = Br/I) as triplet sensitizers, with excitation transfer to 9,10-diphenylanthracene (DPA) and/or 9,10-bis[(triisopropylsilyl)ethynyl]anthracene (TIPS-An) which act as the triplet annihilators. We observe that the upconversion efficiency is five times higher with the combination of both annihilators in a composite system compared to the sum of the individual single-acceptor systems. Our work illustrates the importance of using a composite system of annihilators to enhance TTA upconversion, demonstrated in a perovskite-sensitized system, with promise for a range of potential applications in light-harvesting, biomedical imaging, biosensing, therapeutics, and photocatalysis.

In organic molecules, upon light excitation, electrons from the ground state are excited to the singlet excited state and leave behind positively charged holes, producing singlet excitons that can show efficient radiative recombination.^1^ In contrast, triplet excitons cannot be directly photoexcited and are typically generated from singlet excitons via intersystem crossing. Once formed, returning to the ground state requires a spin-flip, and therefore triplet excitons are typically nonemissive and long-lived. A way to overcome this consists of taking advantage of triplet–triplet annihilation (TTA).^2^ This is a process whereby two triplet excitons from different molecules encounter each other, resulting in one emitter in the ground state and another in the singlet excited state that can undergo fluorescence. The mechanism of TTA was discovered in the 1960s,^3^ and TTA was utilized for photon upconversion in the 2000s,^4,5^ with applications including solar energy,^6,7^ optogenetics,^8,9^ catalysis,^10,11^ lighting,^12,13^ and biomedical imaging.^14,15^ In solar photovoltaics, harvesting sub-bandgap photons by means of upconversion is a path toward overcoming the thermodynamic efficiency limits in single-junction solar cells.^16^ In biomedical imaging, upconverted luminescence can be isolated from the excitation light by using short-pass filters, increasing the signal-to-noise ratio.^17^

A variety of classes of materials have been used as triplet sensitizers to populate the triplet state of organic emitters:^18^ organometallic complexes containing heavy metal atoms, where the triplet state is populated by intersystem crossing;^19,20^ phosphorescent complexes, where spin–orbit coupling enables direct singlet-to-triplet absorption;^21^ lead chalcogenide (PbS and PbSe) nanocrystals;^22−24^ semiconductor quantum dots, which undergo spin dephasing to directly populate the triplet state of the organic annihilators;^25^ and thermally activated delayed fluorescence molecules, where their small exchange energy can lead to efficient intersystem crossing and population of the triplet state.^26−28^ However, each approach has its limitations. For instance, metal–organic complexes have significant exchange energy losses of up to 300 meV. Phosphorescent molecules are in general costly. Lead chalcogenide nanocrystals have poor exciton diffusion lengths, resulting in inefficient exciton transport, which hinders their triplet sensitization ability in films.^29,30^

Recently, halide perovskites have emerged as a class of triplet sensitizers,^29−40^ resulting from the outstanding optoelectronic properties that have put them at the vanguard of emerging photovoltaics^41−44^ and light-emitting devices.^45−47^ These include tunable bandgaps via facile compositional changes, efficient light absorption, long carrier lifetimes and diffusion lengths, low nonradiative recombination, shallow defects, and solution-processability. Additionally, strong spin–orbit coupling due to the abundance of heavy atoms can rapidly spin-mix electrons and holes, providing the possibility of triplet sensitization via separate injection of free electrons and holes into the upconverting organic semiconductor.

Perovskite nanocrystals have also lately received immense attention for optical applications.^48,49^ Kimizuka and co-workers demonstrated the potential of perovskite nanocrystals for photon upconversion.^29^ They used halide-exchanged CsPbBr_3_ nanocrystals as triplet sensitizers, which undergo energy transfer to surface-bound triplet acceptors possessing an amino group, which in turn readily relay triplets to free 9,10-diphenylanthracene (DPA) molecules in solution that undergo TTA. They obtained a low threshold intensity of 25 mW/cm^2^, where the excitation intensity dependence of the upconversion emission intensity changes from quadratic to linear. The maximum TTA efficiency (normalized to 100%) was 1.3%.

Since then, Wu et al. demonstrated triplet energy transfer from perovskite nanocrystals to organic ligands in a series of works using time-resolved photoluminescence and transient absorption studies. They showed that triplet energy transfer can also be mediated by electron transfer.^31^ In addition, they demonstrated that quantum-confined nanocrystals improve the triplet energy transfer efficiency significantly compared to larger, bulk-like nanocrystals. This is because quantum-confinement enables a strong donor–acceptor wave function overlap required for Dexter-type triplet energy transfer. For instance, the energy transfer efficiency rises from 0.006% at 11.2 nm nanocrystals to 99.4% at 3.5 nm quantum-confined nanocrystals.^32^ This approach was adopted to achieve above 10% efficiency for visible-to-ultraviolet upconversion, with 1-naphthalenecarboxylic acid as the organic ligand and 2,5-diphenyloxazole as the emitter.^33^ The efficiency achieved is 2.5 times more than a similar system but using bulk nanocrystals.^34^ Most recently, Koharagi et al. provided a demonstration of TTA-UC from green-to-UV light sensitized by CsPbBr_3_ perovskite nanocrystals.^35^ The triplet energy transmitter was 4-(2-phenyloxazol-5-yl)benzenesulfonate with a sulfonate group, and the emitter was TIPS-naphthalene. The TTA-UC efficiency obtained was 0.014% at an excitation intensity of 16 W/cm^2^. In parallel, Nienhaus et al. have published a series of papers investigating the use of methylammonium formamidinium (MAFA) lead triiodide bulk perovskite to sensitize triplet states in rubrene doped with 1% dibenzotetraphenylperiflanthene (DBP) as the emitter.^36−40^

However, the efficiencies of perovskite-sensitized TTA-UC still often lag behind those of systems sensitized by organic molecules. For example, unlike an efficiency of 2.55% by Askes et al.^50^ or ∼4% by Cao et al.^51^ for platinum(II) octaethylporphyrin (PtOEP) as the triplet sensitizer and DPA as the annihilator, the early demonstration of perovskites as triplet sensitizers using DPA as the annihilator as well obtained an efficiency of only 1.3%.

Here, we report triplet–triplet annihilation in a composite system of annihilators sensitized by perovskite nanocrystals, as a means to obtain significantly enhanced upconversion efficiency. The system design is summarized in Scheme 1, where photoexcitation of the nanocrystals is followed by triplet energy transfer to the organic ligand, then triplet energy transfer to the free emitter molecules in solution, which undergo TTA. Two blue emitters are used for our work: DPA and TIPS-An. The upconversion yield of the composite system (Scheme 1c) is more than five times that of the sum of the single-acceptor systems (Scheme 1a,b). Our work presents a simple and promising approach for highly efficient perovskite-sensitized triplet–triplet annihilation upconversion systems that could be utilized in a range of light-harvesting, biomedical, and photocatalysis applications.

**Scheme 1:**
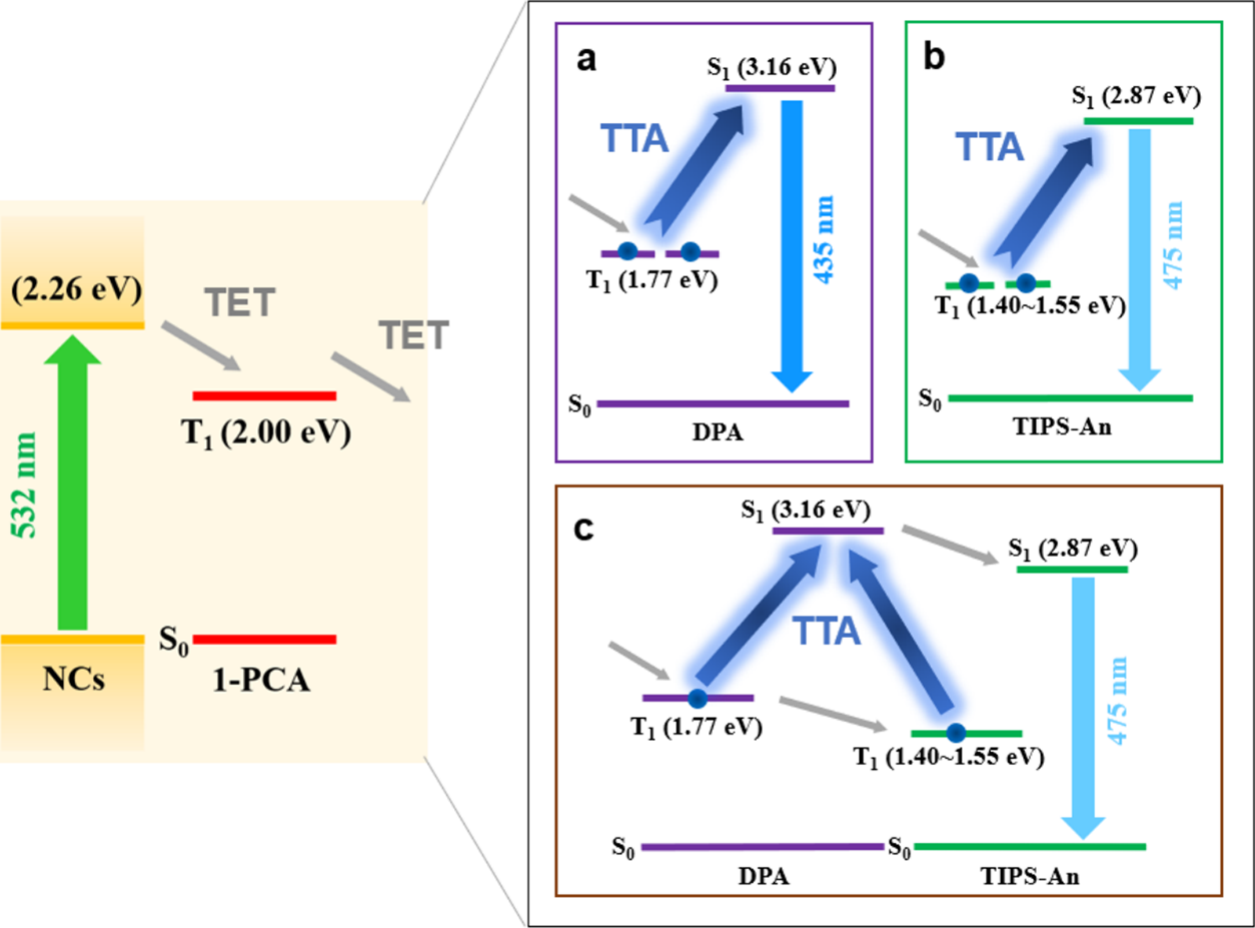
Energetics of the System Design for Green-to-Blue TTA-UC Sensitized by CsPbX_3_ (X = Br/I) Nanocrystals Photoexcitation (green arrow) of the nanocrystals is followed by triplet energy transfer (TET) to the surface-bound 1-PCA. This is then followed by TET to free emitter molecules in solution, which undergo triplet–triplet annihilation (TTA), and finally higher-energy upconverted emission (lighter blue arrows). The free emitter molecules could be (a) DPA, (b) TIPS-An, or (c) both DPA and TIPS-An.

## Results and Discussion

**Figure 1:**
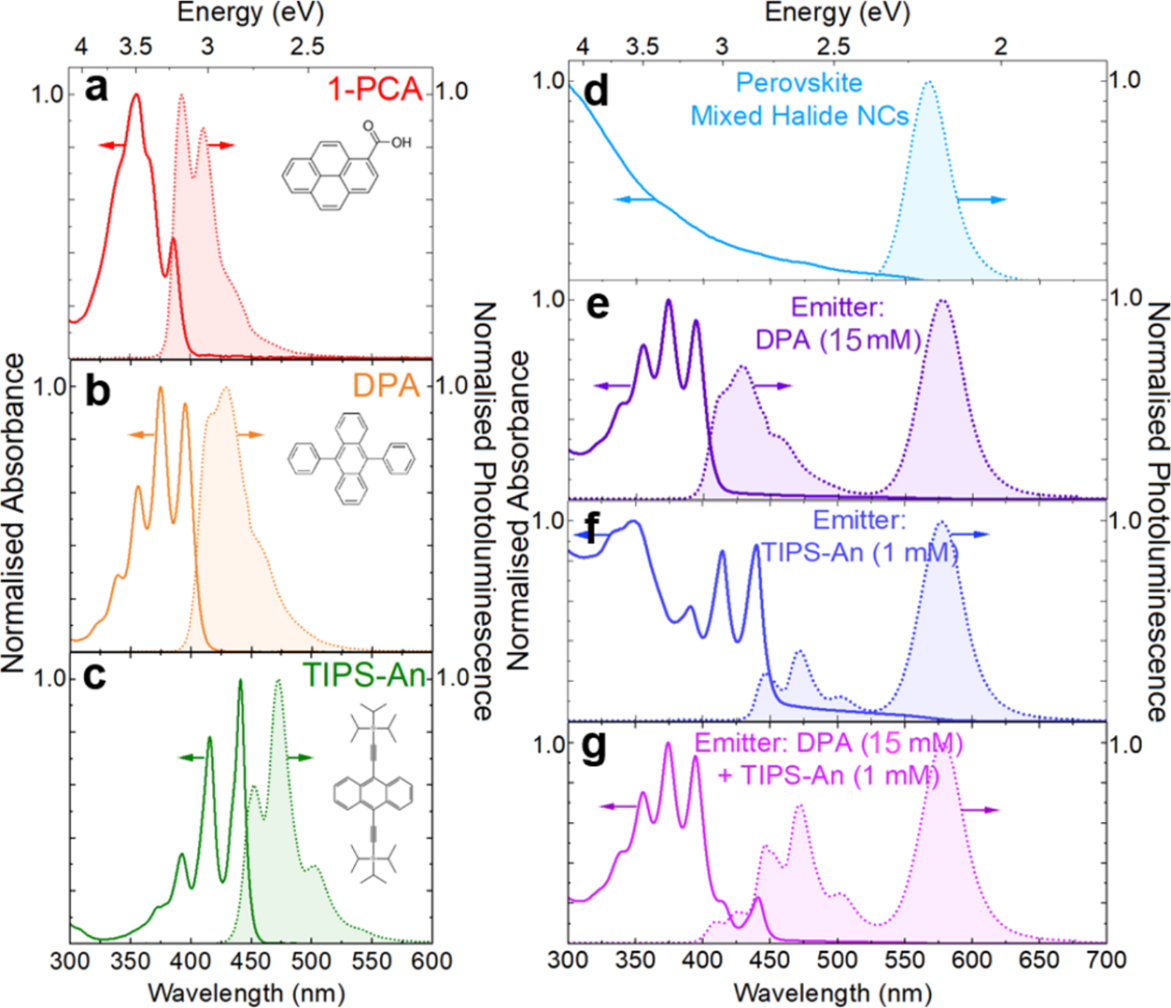
Normalized steady-state absorption (solid lines) and photoluminescence (dashed lines with shadings, excitation 290 nm) of pristine organic molecules (a) 1-PCA, (b) DPA, and (c) TIPS-An, as well as that of (d) pristine mixed halide nanocrystals (5.0 mg/mL), and (e, f) mixed halide nanocrystals (5.0 mg/mL) with surface modification by 1-PCA (0.95 mM) and various emitters (e) DPA, (f) TIPS-An, and (g) both DPA and TIPS-An, in toluene.

We use mixed halide perovskite nanocrystals as the triplet sensitizers. CsPbBr_3_ perovskite nanocrystals with oleic acid and oleylamine as ligands were first synthesized using a hot injection method. PbI_2_ was then added to get the final mixed halide composition of approximately 30% iodide out of total halide by halide substitution, obtaining chemically stable nanocrystals without noticeable halide segregation (see the Methods section for details). Figure 1a–c shows the steady-state absorption and photoluminescence spectra of the pristine organic molecules in our system (1-pyrenecarboxylic acid (1-PCA), DPA, and TIPS-An). Figure 1d–g shows these spectra for the pristine perovskite nanocrystals and the surface-modified perovskite nanocrystals with various emitters in toluene. The pristine perovskite nanocrystals have an absorption onset at ∼550 nm (∼2.26 eV) with a corresponding emission peak at ∼568 nm (Figure 1d). To avoid the long alkyl chains of oleic acid and oleylamine on the pristine nanocrystal surface from hindering triplet energy transfer from the nanocrystals to free emitter molecules in solution by interrupting the short-range Dexter electron transfer,^29^ we use 1-PCA to modify the surface state of perovskite nanocrystals (Figure 1a). 1-PCA was chosen as the ligand given its enhanced quenching efficiency of the perovskite nanocrystals as compared to other ligands considered (Figure S1). TEM measurements show that the size distribution (10.6 ± 2.3 nm) and morphology of the nanocrystals are retained after surface modification with the 1-PCA ligand (Figure S2). XRD measurements also confirm that the cubic perovskite structure has been retained (Figure S3). The triplet energy is transferred from the photoexcited nanocrystals (bandgap, 2.26 eV) to 1-PCA ligands (triplet energy level, 2.00 eV),^52^ and the 1-PCA ligands will pass on the energy from their triplet states to that of free molecules in solution which then emit. The blue emitters used for our work are DPA (triplet energy level of 1.77 eV)^53^ (Figure 1b) and TIPS-An (with a lower triplet energy level between 1.40 and 1.55 eV)^54^ (Figure 1c). These are complementary emitters at both extremes of the blue range of the visible spectrum (the emission of TIPS-An only begins at 425 nm while that of DPA begins at 400 nm), giving us room to differentiate their upconverted signals in the composite system (Figure 1e–g).

**Figure 2:**
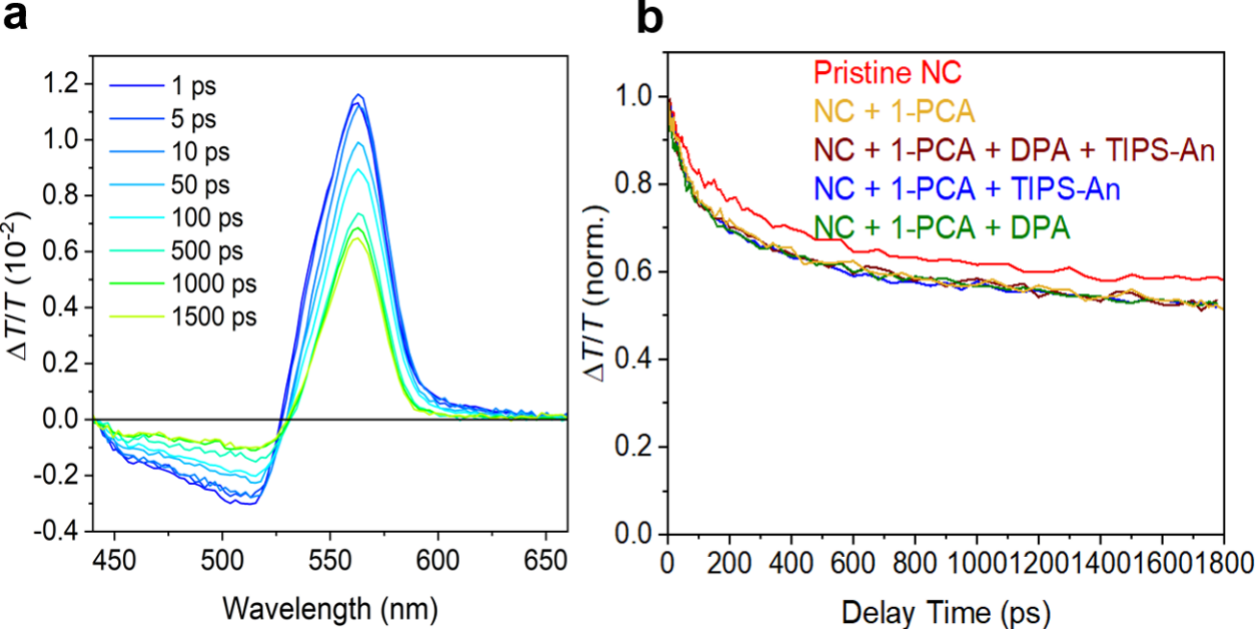
(a) TA spectra of the composite system under a 540 nm pump (up to 1500 ps). (b) TA kinetics of the TTA-UC systems (normalized at peak) for TIPS-An only (blue), DPA only (green), and both DPA and TIPS-An (brown). The control samples are the pure mixed halide nanocrystals (red) and the surface-modified mixed halide nanocrystals (yellow).

To understand how upconversion is promoted by using 1-PCA to mediate triplet energy transfer from the nanocrystals to the organic emitters, we employ transient absorption (TA) spectroscopy to access the early dynamics of the charge carriers upon photoexcitation. Figure 2a shows the TA spectra (up to 1500 ps) of the composite system under a 540 nm pump with a fluence of 3.65 μJ/cm^–2^ (see Figure S4 for the TA spectra of the control and single-acceptor systems). Under this excitation, only the nanocrystals are photoexcited by the pump. The positive signal peaking at ∼560 nm results from the ground-state bleach (GSB) of perovskite nanocrystals, which also reflects the carrier density of the photoexcited nanocrystals. Figure 2b shows the kinetics of the GSB of different systems under the same excitation conditions. The pure nanocrystals (Figure 2b, red) have the longest lifetime corresponding to intrinsic carrier recombination processes. Upon adding 1-PCA, a faster initial decay on the time scale of ∼100 ps appears as compared to the pristine nanocrystals. This demonstrates the quenching of charge carriers from nanocrystals to the 1-PCA ligands (Figure 2b, yellow).^55^ The transfer rate, *k*_et_, is calculated to be 1.4 × 10^8^ s^–1^ by using *k*_et_ = 1/τ_1-PCA_ – 1/τ_NC_, where τ_NC_ (Figure 2b, red curve) and τ_1-PCA_ (Figure 2b, yellow curve) are the initial decay times of the pristine NCs and the NCs with 1-PCA measured using transient absorption. The energy transfer efficiency can be estimated as 1 – τ_1-PCA_/τ_NC_ = 0.12 (see the SI for discussion on low efficiency). Furthermore, this energy transfer is maintained when the blue emitter molecules are added to the ensemble (Figure 2b, brown, blue, and green).

**Figure 3:**
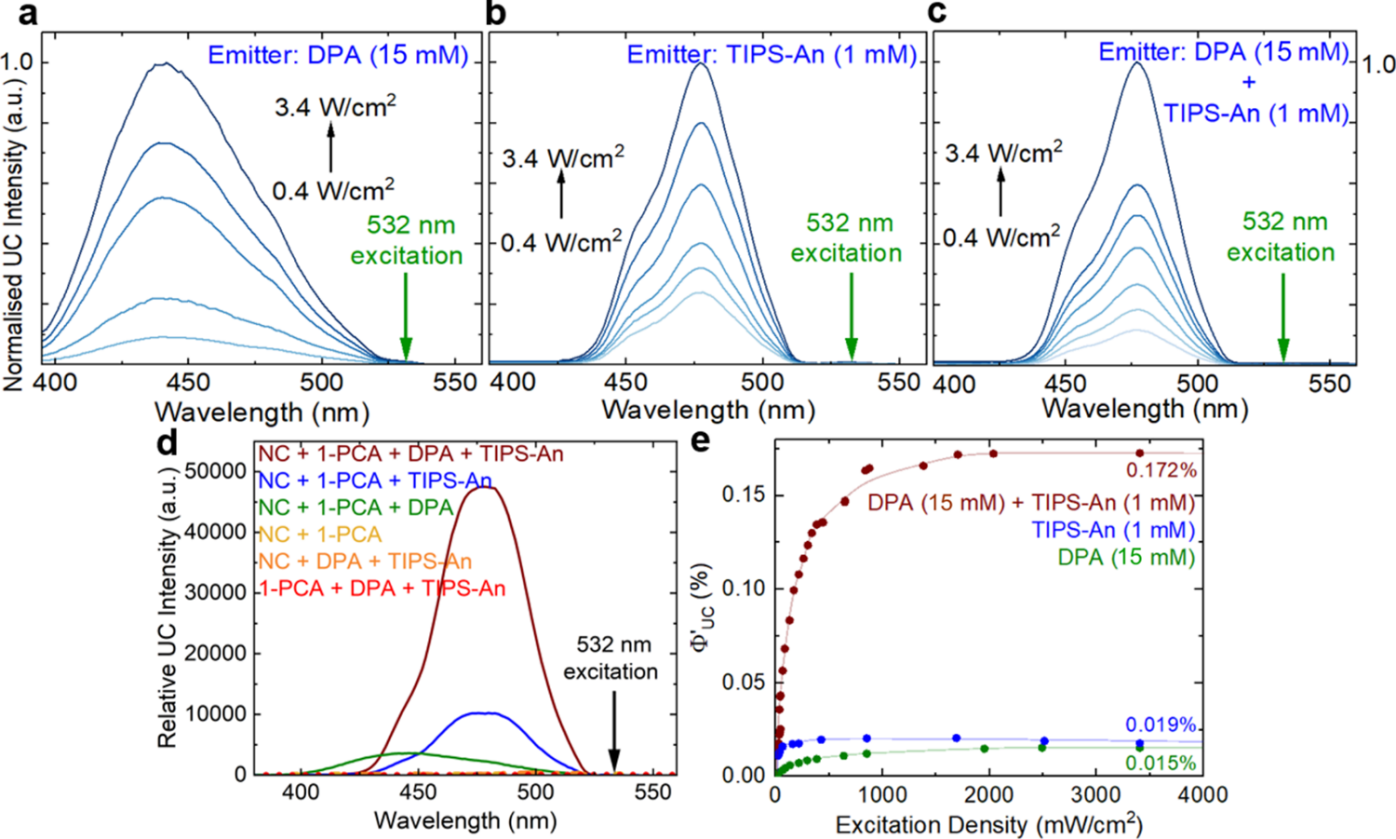
Normalized upconversion emission spectra of surface-modified mixed halide nanocrystals (5.0 mg/mL with 0.95 mM 1-PCA in toluene) with (a) single acceptor of DPA (15 mM), (b) single acceptor of TIPS-An (1 mM), and (c) dual acceptors of DPA (15 mM) and TIPS-An (1 mM). The spectra in (c) closely resemble that of (b). A 532 nm CW laser was used with excitation densities from 0.4 to 3.4 W/cm^2^. A 500 nm short-pass filter was used to remove pump and nanocrystal emissions. (d) Upconversion emission spectra of surface-modified mixed halide nanocrystals (yellow) with TIPS-An only (blue), DPA only (green), and both DPA and TIPS-An (brown), at 430 mW/cm^2^ excitation density, with concentrations identical to (a–c). Control samples are in the absence of emitters (yellow), 1-PCA (orange), or nanocrystals (red) where no upconversion can be observed. (e) Excitation density dependence of Φ’_UC_ for TTA-UC samples (in toluene). The samples were excited with 532 nm CW laser at sufficiently high intensities until the Φ’_UC_ values are saturated.

Figure 3a–c shows the normalized upconversion emission of surface-modified mixed halide nanocrystals with a single acceptor of DPA, single acceptor of TIPS-An, and dual acceptors of DPA and TIPS-An respectively, with excitation densities from 0.4 to 3.4 W/cm^2^. After preliminary optimization, we have chosen the concentrations for TTA to be 15 mM for DPA and 1 mM for TIPS-An (Figure S5). The upconversion emission for DPA peaks at 435 nm, while the upconversion emission for TIPS-An peaks at 475 nm. The upconversion emission from the nested system of DPA and TIPS-An closely resembles the emission spectrum of TIPS-An, indicating that the upconversion emission originates from TIPS-An only, instead of DPA or a mixture of both. As DPA is much more concentrated than TIPS-An, we propose that DPA acts as a mediator for TIPS-An.

Figure 3d shows the relative intensity of upconversion emissions of the three systems under study, for the same excitation intensity of 430 mW/cm^2^ (excitation wavelength at 532 nm). As mentioned, the intensity of upconversion emission for the nested system (Figure 3d, brown) is much higher than the linear sum of the emissions from the single-acceptor systems (Figure 3d, blue and green). Control experiments with identical concentrations were performed to confirm that the emissions result from the upconversion of the composite system. No upconversion emission is observed in the absence of organic emitters (Figure 3d, yellow). Upconversion emission is also not detected for a mixture of the mixed halide nanocrystals and DPA/TIPS-An without surface modification by 1-PCA (Figure 3d, orange). This result confirms that the surface modification by 1-PCA is critical for triplet energy transfer to occur from the nanocrystals to 1-PCA, and onward to the free emitter molecules in solution. Finally, no upconversion emission is observed from a solution of 1-PCA, DPA, and TIPS-An without perovskite nanocrystals (Figure 3d, red). This shows that the triplet excited state of organic molecules is populated by direct excitation of the perovskite nanocrystals.

To discard changes in absorption affecting the relative upconversion signals, we measured the quantum yield of the upconversion emissions, as shown in Figure 3e (see Methods for details of the TTA efficiency calculations). Since the maximum yield of a bimolecular TTA-UC process is 50%, we normalize the upconversion quantum yield against 100% and denote it by Φ’_UC_. With the increase of excitation intensity, Φ’_UC_ gradually increases and becomes saturated. The highest upconversion quantum yield of the nested system is 0.172% (Figure 3e, brown), much higher than the linear sum of both single-acceptor systems (0.019%, blue and 0.015%, green, respectively). We also performed direct excitation of singlet states using a 400 nm pump for the nested system. We observed an approximate linear sum of the emissions of the single-acceptor systems (Figure S6), confirming the distinct nature of the nonlinear enhancement of TTA efficiency in the nested system via the mediator.

**Figure 4:**
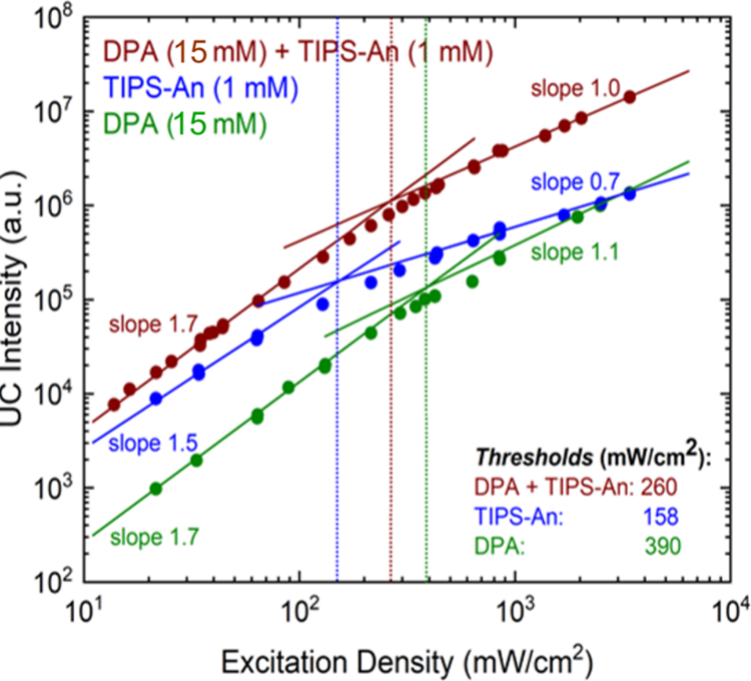
Excitation density dependence of integrated upconversion emission intensity for TTA-UC samples (in toluene). Solid lines show the linear fitting with slopes indicated. The intersection gives the threshold excitation density, denoted by vertical dashed lines.

Figure 4 shows the excitation density dependence of the integrated upconversion emission of all of the TTA-UC systems. The TTA-UC emission intensity shows quadratic dependence at low excitation intensity and linear dependence at high excitation intensity. The quadratic regime is dominated by radiative and nonradiative decay of emitter triplets, whereas TTA becomes the main deactivation channel for the acceptor triplet states at the linear regime.^56^ The threshold where the quadratic-to-linear transition takes place is 390 mW/cm^2^ for DPA, 158 mW/cm^2^ for TIPS-An, and 260 mW/cm^2^ for DPA/TIPS-An.

Having demonstrated the nonlinear enhancement of TTA efficiency in the composite system, we turn our attention to the possible mechanistic pathways. The 1-PCA ligands do not have optical signals in a wavelength region distinct from the annihilator molecules—either ground-state bleach (GSB, bleaching of S_0_ to S_n_) or excited-state absorption (absorption from S_1_ to S_n_)—so it would be hard to directly probe carrier density change in the 1-PCA ligands. We therefore rely on the time-dependent carrier densities in the perovskite nanocrystals from TA measurements for insights. A comparison of the GSB kinetics between different samples in Figure 2b shows that all samples with additives (1-PCA, DPA, TIPS-An) quench the pristine nanocrystals to the same degree, as evidenced by the similar initial decay of the GSB. This indicates that the addition of DPA or TIPS-An would not dramatically change the carrier extraction from the nanocrystals. This is consistent with Figure 4 where the threshold of the DPA + TIPS-An system (brown) lies between that of the individual DPA (green) and the TIPS-An systems (blue). In addition, we note that the observed enhancement cannot be due to absorption differences alone, due to the same concentration of perovskite nanocrystals in all systems. We now examine possible pathways for this enhancement (Figure S7). In mechanism (I), triplet excited states from two DPA molecules interact to produce a singlet excited state of DPA, which immediately transfers its energy to the singlet of TIPS-An, i.e., T_1_ (DPA) + T_1_ (DPA) → S_1_ (DPA) → S_1_ (TIPS-An). In mechanism (II), the lower T_1_ (TIPS-An) as compared to T_1_ (DPA) facilitates an efficient energy cascade of triplets via T_1_ (DPA) to T_1_ (TIPS-An), leading to enhanced concentration of triplet excited states in TIPS-An. Two triplet excited states from two TIPS-An molecules then interact to produce a singlet excited state of TIPS-An.

The relatively lower threshold of the composite system compared with the DPA-only system in Figure 4 shows that it is unlikely that the TTA in the composite system is only related to DPA. This indicates that mechanism (I) probably plays a less significant role in accounting for the enhancement. Additionally, the degree of quenching is very similar for surface-modified nanocrystals with different emitters in Figure 2b (green for DPA; blue for TIPS-An; brown for DPA and TIPS-An), indicating that the addition of an emitter does not significantly promote the process of energy transfer from nanocrystals to organic molecules. It is therefore unlikely that the mere addition of DPA, behaving as a triplet intermediary between 1-PCA and TIPS-An, would, on its own, lead to the nonlinear enhancement of TTA between TIPS-An molecules. Furthermore, the photoluminescence quantum yield of TIPS-An is lower than that of DPA. Hence, this indicates that mechanism (II) also probably plays a diminished role in accounting for the enhancement.

There is a third mechanism that could be consistent with the above results: a composite TTA process between a DPA molecule and a TIPS-An molecule. This is illustrated in Scheme 1c, where a triplet excited state from DPA and a triplet excited state from TIPS-An undergo TTA to produce a bright singlet state. The singlet of DPA S_1_ (DPA, 3.16 eV) and the singlet of TIPS-An S_1_ (TIPS-An, 2.87 eV) can be reached from T_1_ (DPA, 1.77 eV) + T_1_ (TIPS-An, 1.40–1.55 eV). As S_1_ (DPA) is resonant with T_1_ (DPA) + T_1_ (TIPS-An), upconversion to S_1_ (DPA) is more likely to happen. The excitation then undergoes ultrafast energy transfer from S_1_ (DPA) to S_1_ (TIPS-An), leading to the observation of the lower-energy PL from TIPS-An dominating the composite system’s emission spectrum. It may also be possible for T_1_ (DPA) and T_1_ (TIPS-An) to undergo TTA to populate S_1_ (TIPS-An) directly, although we believe this is less likely due to the resonance of S_1_ (DPA) with T_1_ (DPA) + T_1_ (TIPS-An) instead. Scheme 1b represents the underlying TTA-UC arising from direct triplet transfer from 1-PCA to T_1_ (TIPS-An) in the nested system. We provide a mathematical model to provide insights for why we believe Scheme 1c may lead to an overall higher TTA-UC emission intensity, taking into consideration that both the emitters DPA and TIPS-An individually have TTA-UC efficiencies close to their theoretical limits when used in other systems (see Section 2 in the SI). It is however challenging to obtain direct time-resolved evidence due to the low overall upconversion efficiencies (see Section 3 in the SI for further discussion), and further work utilizing more efficient TTA systems will be valuable and the subject of our future studies.

Finally, we comment on similarities and differences between our work and others (Figure S8). Cao et al.^51^ demonstrated a synergistic effect in phosphor-sensitized TTA-UC systems arising from TTA upconversion between triplet acceptors of different types, which they term “hetero-TTA”, and the upconversion signals from both acceptors are observed (Figure S8a,b). We also note a recent report by Yurash et al.^57^ which utilized a three-component system employing thermally activated delayed fluorescence sensitizers, achieving a doubling of upconversion efficiency, with upconversion observed only from one emitter (Figure S8c,d). Our work in fact combines features of both reports, where we employ a composite system of annihilators, and we observe upconversion signals purely from TIPS-An in this composite system. Our system design has generated a 5-fold enhancement compared to the sum of individual systems. In addition, our work represents the successful report of significant enhanced TTA-UC in a perovskite-sensitized system involving a mix of annihilators. Our work confirms the generality of multiacceptor systems in enhancing TTA-UC efficiencies, and will be highly applicable to other upconversion systems employing different sensitizers. The higher efficiencies to be gained, for example, using quantum-confined nanocrystals (see Figure S9 and SI for further discussion), will further exemplify the detailed photophysical processes and limits of such enhancements, in turn bringing further exciting advances to these highly enhanced photon upconversion systems.

## Conclusions

We report the observation of significantly enhanced TTA upconversion in a composite system comprising mixed halide perovskite nanocrystals as the triplet sensitizers, 1-PCA ligands to transfer triplet energy to the triplet states of free molecules in solution, and both DPA and TIPS-An as the annihilators. The highest upconversion quantum yield of the composite system is 0.172%, much higher than the linear sum of both single-acceptor systems (0.019 and 0.015%). The upconversion emission of the composite system originates from TIPS-An only. We obtain the threshold values of the quadratic-to-linear transition and the transient absorption kinetics, and discuss three possible mechanistic pathways of TTA-UC in the composite system. Our results suggest that the enhanced efficiency may arise from an energy resonance between the sum of the triplet excited states of DPA and TIPS-An with the singlet excited state of DPA. This resonance is followed by ultrafast energy transfer to the emissive singlet state of TIPS-An, leading to upconversion emission dominated by TIPS-An. Our work demonstrates the distinct opportunities and benefits that a composite system of annihilators provides to significantly increase the performance of TTA upconversion systems, utilized with mixed halide perovskite nanocrystals as triplet sensitizers, and highlights the need for further in-depth photophysical investigations in more efficient systems. The simple approach here will find practical use in a range of applications such as light-harvesting, biosensing, therapeutics, biomedical imaging, and photocatalysis.

## Methods

### Materials

All reagents and solvents for synthesis and sample preparation were used as received. PbBr_2_, oleic acid, oleylamine, 1-pyrenecarboxylic acid (1-PCA), 9-anthracenecarboxylic acid (9-ACA), 1-naphthalenecarboxylic acid (1-NCA), 9,10-diphenylanthracene (DPA), and 9,10-bis[(triisopropylsilyl)ethynyl]anthracene (TIPS-An) were purchased from Sigma-Aldrich.

### Nanocrystal Synthesis

The synthesis of CsPbBr_2.1_I_0.9_ nanocrystals was performed by modifying the procedure by Kovalenko et al.^58^ Cesium carbonate (Cs_2_CO_3_, 0.814 g, 2.5 mmol), oleic acid (OA, 2.5 mL, 7.5 mmol), and octadecene (ODE, 30 mL) were added into a three-neck flask, degassed under vacuum for 1 h at 110 °C, and then heated under N_2_ to 150 °C until all of the Cs_2_CO_3_ dissolved and reacted with OA. Oleylamine (OLA, 1.5 mL, 4.5 mmol), OA (1.5 mL, 4.5 mmol), ODE (15 mL), and PbBr_2_ (0.207 g) were added into another three-neck flask and degassed for 1 h at 110 °C. After complete solubilization of PbBr_2_ salt, a clear solution was formed. Then, the temperature was raised to the reaction temperature (170 °C) under N_2_ and the Cs-oleate precursor (1.2 mL) was quickly injected into the PbBr_2_ solution. After 5 s, the reaction mixture was cooled by an ice-cold water bath to room temperature (25 °C), followed by centrifuging at 12,000 rpm (16728 RCF) for 5 min. The supernatant was discarded, and the nanocrystals were redispersed in toluene. For mixed halide nanocrystals, the PbI_2_ solution prepared by adding 0.2 mmol of PbI_2_ into 10 mL of toluene with 0.2 mL of OA and OLA as ligands was added into the CsPbBr_3_ nanocrystals. The mixture was stirred at 40 °C until the anion exchange was fully complete. The obtained mixed halide nanocrystals were purified by centrifuge at 3000 rpm (1045 RCF), without antisolvent.

### Sample Preparation

For surface modification, equal volumes of mixed halide nanocrystals solution and 1-pyrenecarboxylic acid (1-PCA) in toluene were added, to obtain final solution concentrations of 5.0 mg/mL mixed halide nanocrystals and 0.95 mM 1-PCA respectively. The anhydrous toluene solvent was not degassed, and used as received from Sigma-Aldrich. The mixture was stirred at room temperature for 6 h at 100 rpm in capped vials further sealed with parafilm. The obtained mixture was filtered through a 0.45 μm PTFE syringe filter. 15 mM DPA, 1 mM TIPS-An, or both 15 mM DPA and 1 mM TIPS-An were then added and mixed thoroughly before the solution was transferred into a 1 mm path length quartz cuvette which was sealed with a PTFE cap and parafilm. The concentration of 15 mM DPA or 1 mM TIPS-An for single-acceptor systems was chosen after preliminary optimization (Figure S5). The entire procedure, except stirring, was conducted in a N_2_-filled glovebox.

### Steady-State Absorption and Photoluminescence Spectroscopy

Absorption spectra were measured using a Shimadzu UV3600-Plus UV–vis–NIR spectrophotometer. Photoluminescence spectra were measured on an Edinburgh Instruments FLS980 fluorimeter with a 450 W continuous xenon arc lamp. The samples were filled in a 1 mm path length quartz cuvette.

### Measurement of Upconversion Spectra with Integrating Sphere

Upconverison spectra were measured in an integrating sphere with a detector constructed with a spectroscopy camera (Andor iDus DU420A Si detector, ANDOR) coupled to a spectrograph (Shamrock SR303i, ANDOR) and samples filled in a 1 mm path length quartz cuvette excited by a 532 nm CW laser (Thorlabs) passing through an FES0500 short-pass filter (Thorlabs).

### Quantum Efficiency Measurement of Mixed Halide Nanocrystals Solution with Integrating Sphere

PLQE measurements were made following the procedure of de Mello et al.^59^ Temperature- and current-controlled laser diodes (Thorlabs) were used to generate stable laser beams with wavelength 405 or 520 nm. After attenuation to the desired intensity, these were aligned through a small hole, onto samples suspended, in a Spectralon-coated integrating sphere (Newport 819C-SL-5.3) modified with a custom baffle extension. Light from the experiment was collected using an optical fiber connected to an Andor Kymera 328i spectrograph, and spectra were recorded using a Si-CCD (Andor iDus 420).

### Quantum Efficiency Measurement of TTA-UC Samples with Relative Method

The normalized upconversion quantum yield Φ’_UC_ was calculated indirectly with the following equation:^60^
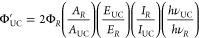
where Φ*_R_* is the quantum yield of the reference, *A* is the absorption at the excitation wavelength, *E* is the integrated photoluminescence spectral profile, *I* is the light intensity at the excitation wavelength, and *h*ν is the energy of excitation photons. The subscripts UC and *R* denote the upconversion and reference samples respectively. The mixed halide nanocrystals in deaerated toluene solution with an excitation of 405 nm (Φ*_R_* = 0.290) and 520 nm (Φ*_R_* = 0.353) were used as the reference, and the upconversion efficiency was calculated from the average of both independent values. The factor of 2 arises because the maximum yield of a bimolecular TTA-UC process is 50% since two photons are required to generate one upconverted photon. Therefore, we normalize the upconversion quantum yield to 100% and denote it by Φ’_UC_. This allows comparison with other recent works in perovskite-sensitized TTA-UC systems.^29,33,34,39,61^ We acknowledge the push in the field to report unnormalized TTA-UC efficiencies.^62^ Without normalization, the upconversion quantum yields would be half that reported above in the main text. The TTA efficiency could not be obtained directly because a 500 nm short-pass filter was necessary to prevent the saturation of the detector.

### Transmission Electron Microscopy

Transmission electron microscopy samples were prepared by putting a small drop of mixed halide nanocrystals solution onto a carbon-coated copper grid in ambient air. Transmission electron microscopy images were recorded using an FEI Tecnai F20 (200 kV).

### X-ray Diffraction

XRD measurements were performed using a Bruker X-ray D8 Advance diffractometer with Cu Kα radiation (λ = 1.5406 Å). Diffractograms were collected within an angular range of 5° ≤ 2θ ≤ 50° and with Δ2θ = 0.02453° steps.

### Time-Resolved Photoluminescence (iCCD)

Time-resolved photoluminescence spectra were attempted with an electrically gated intensified charge-coupled device (iCCD) camera (AndoriStar DH740 CCI-010) connected to a grating spectrometer (Andor SR303i).

### Time-Correlated Single Photon Counting (TCSPC)

TCSPC plots were measured on an Edinburgh Instruments FLS1000 fluorimeter equipped with a 450 W continuous xenon arc lamp. A picosecond pulsed diode laser at 505.8 nm (EPL-510) was used (repetition rate of 5 MHz at a fluence of 16.2 nJ/cm^2^/pulse), which was connected to the spectrometer using a coupling flange.

### Transient Absorption Spectroscopy

The output of a Ti:sapphire amplifier system (Spectra Physics Solstice Ace) operating at 1 kHz and generating ∼100 fs pulses was split into the pump and probe beam paths. The 540 nm pump pulses were created by sending the 800 nm fundamental beam of the Solstice Ace into a home-built noncollinear optical parametric amplifier (NOPA). The pump goes through a 540 nm bandpass filter and then was blocked by a chopper wheel rotating at 500 Hz. The ultraviolet–visible broadband beam (330–700 nm) was generated by focusing the 800 nm fundamental beam onto a CaF_2_ crystal (Eksma Optics, 5 mm) connected to a digital motion controller (Mercury C-863 DC Motor Controller) after passing through a mechanical delay stage (Thorlabs DDS300-E/M). The transmitted pulses were collected with a monochrome line scan camera (JAI SW-4000M-PMCL, spectrograph: Andor Shamrock SR-163) with collected data fed straight into the computer.

## Data Availability

The data that support the findings of this study are openly available in Apollo, the University of Cambridge Repository, at 10.17863/CAM.108423.

## Supporting Information Available

TEM, XRD, TCSPC, additional TA data, additional PL data, alternative mechanisms discussed, and additional comments (PDF)

## References

[ref1] Xie W.; Tian L.; Wu K.; Guo B.; Gong J. R. Understanding and modulating exciton dynamics of organic and low-dimensional inorganic materials in photo(electro)catalysis. J. Catal. 2021, 395, 91–104. 10.1016/j.jcat.2020.12.030.

[ref2] Schloemer T.; Narayanan P.; Zhou Q.; Belliveau E.; Seitz M.; Congreve D. N. Nanoengineering Triplet–Triplet Annihilation Upconversion: From Materials to Real-World Applications. ACS Nano 2023, 17 (4), 3259–3288. 10.1021/acsnano.3c00543. 36800310

[ref3] Parker C. A.; Hatchard C. G. Delayed Fluorescence from Solutions of Anthracene and Phenanthrene. Proc. R. Soc. London, Ser. A 1962, 269 (1339), 574–584. 10.1098/rspa.1962.0197.

[ref4] Singh-Rachford T. N.; Castellano F. N. Photon upconversion based on sensitisedsensitized triplet–triplet annihilation. Coord. Chem. Rev. 2010, 254 (21), 2560–2573. 10.1016/j.ccr.2010.01.003.

[ref5] Baluschev S.; Miteva T.; Yakutkin V.; Nelles G.; Yasuda A.; Wegner G. Up-Conversion Fluorescence: Noncoherent Excitation by Sunlight. Phys. Rev. Lett. 2006, 97 (14), 143903 10.1103/PhysRevLett.97.143903. 17155253

[ref6] Gray V.; Dzebo D.; Abrahamsson M.; Albinsson B.; Moth-Poulsen K. Triplet–triplet annihilation photon-upconversion: towards solar energy applications. Phys. Chem. Chem. Phys. 2014, 16 (22), 10345–10352. 10.1039/C4CP00744A. 24733519

[ref7] Sheng W.; Yang J.; Li X.; Liu G.; Lin Z.; Long J.; Chen Y. Tremendously enhanced photocurrent enabled by triplet–triplet annihilation up-conversion for high-performance perovskite solar cells. Energy Environ. Sci. 2021, 14 (6), 3532–3541. 10.1039/D1EE00631B.

[ref8] Sasaki Y.; Oshikawa M.; Bharmoria P.; Kouno H.; Hayashi-Takagi A.; Sato M.; Ajioka I.; Yanai N.; Kimizuka N. Near-Infrared Optogenetic Genome Engineering Based on Photon-Upconversion Hydrogels. Angew. Chem., Int. Ed. 2019, 58 (49), 17827–17833. 10.1002/anie.201911025. 31544993

[ref9] Meir R.; Hirschhorn T.; Kim S.; Fallon K. J.; Churchill E. M.; Wu D.; Yang H. W.; Stockwell B. R.; Campos L. M. Photon Upconversion Hydrogels for 3D Optogenetics. Adv. Funct. Mater. 2021, 31, 2010907 10.1002/adfm.202010907. PMC13480420 42610119

[ref10] Huang L.; Wu W.; Li Y.; Huang K.; Zeng L.; Lin W.; Han G. Highly Effective Near-Infrared Activating Triplet–Triplet Annihilation Upconversion for Photoredox Catalysis. J. Am. Chem. Soc. 2020, 142 (43), 18460–18470. 10.1021/jacs.0c06976. 33074671

[ref11] Hwang S. Y.; Song D.; Seo E. J.; et al. Triplet–triplet annihilation-based photon-upconversion to broaden the wavelength spectrum for photobiocatalysis. Sci. Rep. 2022, 12, 9397 10.1038/s41598-022-13406-8. 35672399 PMC9174481

[ref12] Graf von Reventlow L.; Bremer M.; Ebenhoch B.; Gerken M.; Schmidt T. W.; Colsmann A. An add-on organic green-to-blue photon-upconversion layer for organic light emitting diodes. J. Mater. Chem. C 2018, 6 (15), 3845–3848. 10.1039/C7TC05649D.

[ref13] Lim H.; Cheon H. J.; Lee G. S.; Kim M.; Kim Y.-H.; Kim J.-J. Enhanced Triplet–Triplet Annihilation of Blue Fluorescent Organic Light-Emitting Diodes by Generating Excitons in Trapped Charge-Free Regions. ACS Appl. Mater. Interfaces 2019, 11 (51), 48121–48127. 10.1021/acsami.9b15303. 31774270

[ref14] Wohnhaas C.; Mailänder V.; Dröge M.; Filatov M. A.; Busko D.; Avlasevich Y.; Baluschev S.; Miteva T.; Landfester K.; Turshatov A. Triplet–Triplet Annihilation Upconversion Based Nanocapsules for Bioimaging Under Excitation by Red and Deep-Red Light. Macromol. Biosci. 2013, 13, 1422–1430. 10.1002/mabi.201300149. 23868857

[ref15] Liu Q.; Xu M.; Yang T.; Tian B.; Zhang X.; Li F. Highly Photostable Near-IR-Excitation Upconversion Nanocapsules Based on Triplet–Triplet Annihilation for in Vivo Bioimaging Application. ACS Appl. Mater. Interfaces 2018, 10 (12), 9883–9888. 10.1021/acsami.7b17929. 29425018

[ref16] Nattestad A.; Cheng Y. Y.; MacQueen R. W.; Schulze T. F.; Thompson F. W.; Mozer A. J.; Fückel B.; Khoury T.; Crossley M. J.; Lips K.; Wallace G. G.; Schmidt T. W. Dye-Sensitized Solar Cell with Integrated Triplet–Triplet Annihilation Upconversion System. J. Phys. Chem. Lett. 2013, 4 (12), 2073–2078. 10.1021/jz401050u. 26283255

[ref17] Askes S. H. C.; Bonnet S. Solving the oxygen sensitivity of sensitized photon upconversion in life science applications. Nat. Rev. Chem. 2018, 2 (12), 437–452. 10.1038/s41570-018-0057-z.

[ref18] Yanai N.; Kimizuka N. New Triplet Sensitization Routes for Photon Upconversion: Thermally Activated Delayed Fluorescence Molecules, Inorganic Nanocrystals, and Singlet-to-Triplet Absorption. Acc. Chem. Res. 2017, 50 (10), 2487–2495. 10.1021/acs.accounts.7b00235. 28930435

[ref19] Singh-Rachford T. N.; Castellano F. N. Pd(II) Phthalocyanine-Sensitized Triplet–Triplet Annihilation from Rubrene. J. Phys. Chem. A 2008, 112 (16), 3550–3556. 10.1021/jp7111878. 18336014

[ref20] Singh-Rachford T. N.; Castellano F. N. Triplet Sensitized Red-to-Blue Photon Upconversion. J. Phys. Chem. Lett. 2010, 1 (1), 195–200. 10.1021/jz900170m.

[ref21] Amemori S.; Sasaki Y.; Yanai N.; Kimizuka N. Near-Infrared-to-Visible Photon Upconversion Sensitized by a Metal Complex with Spin-Forbidden yet Strong S0–T1 Absorption. J. Am. Chem. Soc. 2016, 138 (28), 8702–8705. 10.1021/jacs.6b04692. 27354325

[ref22] Huang Z.; Li X.; Mahboub M.; Hanson K. M.; Nichols V. M.; Le H.; Tang M. L.; Bardeen C. J. Hybrid Molecule–Nanocrystal Photon Upconversion Across the Visible and Near-Infrared. Nano Lett. 2015, 15 (8), 5552–5557. 10.1021/acs.nanolett.5b02130. 26161875

[ref23] Wu M.; Congreve D. N.; Wilson M. W. B.; Jean J.; Geva N.; Welborn M.; Van Voorhis T.; Bulović V.; Bawendi M. G.; Baldo M. A. Solid-state infrared-to-visible upconversion sensitized by colloidal nanocrystals. Nat. Photonics 2016, 10 (1), 31–34. 10.1038/nphoton.2015.226.

[ref24] Okumura K.; Mase K.; Yanai N.; Kimizuka N. Employing Core-Shell Quantum Dots as Triplet Sensitizers for Photon Upconversion. Chem. - Eur. J. 2016, 22 (23), 7721–7726. 10.1002/chem.201600998. 27121225

[ref25] Nienhaus L.; Wu M.; Bulović V.; Baldo M. A.; Bawendi M. G. Using lead chalcogenide nanocrystals as spin mixers: a perspective on near-infrared-to-visible upconversion. Dalton Trans. 2018, 47 (26), 8509–8516. 10.1039/C8DT00419F. 29493697

[ref26] Nakanotani H.; Higuchi T.; Furukawa T.; Masui K.; Morimoto K.; Numata M.; Tanaka H.; Sagara Y.; Yasuda T.; Adachi C. High-efficiency organic light-emitting diodes with fluorescent emitters. Nat. Commun. 2014, 5 (1), 4016 10.1038/ncomms5016. 24874292

[ref27] Wu T. C.; Congreve D. N.; Baldo M. A. Solid state photon upconversion utilizing thermally activated delayed fluorescence molecules as triplet sensitizer. Appl. Phys. Lett. 2015, 107 (3), 031103 10.1063/1.4926914.

[ref28] Yanai N.; Kozue M.; Amemori S.; Kabe R.; Adachi C.; Kimizuka N. Increased vis-to-UV upconversion performance by energy level matching between a TADF donor and high triplet energy acceptors. J. Mater. Chem. C 2016, 4 (27), 6447–6451. 10.1039/C6TC01816E.

[ref29] Mase K.; Okumura K.; Yanai N.; Kimizuka N. Triplet sensitization by perovskite nanocrystals for photon upconversion. Chem. Commun. 2017, 53 (59), 8261–8264. 10.1039/C7CC03087H. 28627558

[ref30] VanOrman Z. A.; Drozdick H. K.; Wieghold S.; Nienhaus L. Bulk halide perovskites as triplet sensitizers: progress and prospects in photon upconversion. J. Mater. Chem. C 2021, 9 (8), 2685–2694. 10.1039/D1TC00245G.

[ref31] Luo X.; Liang G.; Han Y.; Li Y.; Ding T.; He S.; Liu X.; Wu K. Triplet Energy Transfer from Perovskite Nanocrystals Mediated by Electron Transfer. J. Am. Chem. Soc. 2020, 142 (25), 11270–11278. 10.1021/jacs.0c04583. 32479073

[ref32] Luo X.; Lai R.; Li Y.; Han Y.; Liang G.; Liu X.; Ding T.; Wang J.; Wu K. Triplet Energy Transfer from CsPbBr3 Nanocrystals Enabled by Quantum Confinement. J. Am. Chem. Soc. 2019, 141 (10), 4186–4190. 10.1021/jacs.8b13180. 30817139

[ref33] He S.; Luo X.; Liu X.; Li Y.; Wu K. Visible-to-Ultraviolet Upconversion Efficiency above 10% Sensitized by Quantum-Confined Perovskite Nanocrystals. J. Phys. Chem. Lett. 2019, 10 (17), 5036–5040. 10.1021/acs.jpclett.9b02106. 31411888

[ref34] Okumura K.; Yanai N.; Kimizuka N. Visible-to-UV Photon Upconversion Sensitized by Lead Halide Perovskite Nanocrystals. Chem. Lett. 2019, 48 (11), 1347–1350. 10.1246/cl.190473.

[ref35] Koharagi M.; Harada N.; Okumura K.; Miyano J.; Hisamitsu S.; Kimizuka N.; Yanai N. Green-to-UV photon upconversion enabled by new perovskite nanocrystal-transmitter-emitter combination. Nanoscale 2021, 13 (47), 19890–19893. 10.1039/D1NR06588B. 34846408

[ref36] Wieghold S.; Nienhaus L. Precharging Photon Upconversion: Interfacial Interactions in Solution-Processed Perovskite Upconversion Devices. J. Phys. Chem. Lett. 2020, 11 (3), 601–607. 10.1021/acs.jpclett.9b03596. 31894692

[ref37] Wieghold S.; Bieber A. S.; VanOrman Z. A.; Nienhaus L. Influence of Triplet Diffusion on Lead Halide Perovskite-Sensitized Solid-State Upconversion. J. Phys. Chem. Lett. 2019, 10 (13), 3806–3811. 10.1021/acs.jpclett.9b01526. 31244265

[ref38] Bieber A. S.; VanOrman Z. A.; Wieghold S.; Nienhaus L. Perovskite-sensitized upconversion bingo: Stoichiometry, composition, solvent, or temperature?. J. Chem. Phys. 2020, 153 (8), 084703 10.1063/5.0021973. 32872865

[ref39] Wang L.; Yoo J. J.; Lin T.-A.; Perkinson C. F.; Lu Y.; Baldo M. A.; Bawendi M. G. Interfacial Trap-Assisted Triplet Generation in Lead Halide Perovskite Sensitized Solid-State Upconversion. Adv. Mater. 2021, 33 (27), 2100854 10.1002/adma.202100854. 34048075

[ref40] VanOrman Z. A.; Lackner J.; Wieghold S.; Nienhaus K.; Nienhaus G. U.; Nienhaus L. Efficiency of bulk perovskite-sensitized upconversion: Illuminating matters. Appl. Phys. Lett. 2021, 118 (20), 203903 10.1063/5.0050185.

[ref41] Lee M. M.; Teuscher J.; Miyasaka T.; Murakami T. N.; Snaith H. J. Efficient Hybrid Solar Cells Based on Meso-Superstructured Organometal Halide Perovskites. Science 2012, 338 (6107), 643–647. 10.1126/science.1228604. 23042296

[ref42] Liu M.; Johnston M.; Snaith H. Efficient planar heterojunction perovskite solar cells by vapour deposition. Nature 2013, 501, 395–398. 10.1038/nature12509. 24025775

[ref43] Eperon G. E.; Stranks S. D.; Menelaou C.; Johnston M. B.; Herz L. M.; Snaith H. J. Formamidinium lead trihalide: a broadly tunable perovskite for efficient planar heterojunction solar cells. Energy Environ. Sci. 2014, 7 (3), 982–988. 10.1039/c3ee43822h.

[ref44] Green M.; Ho-Baillie A.; Snaith H. The emergence of perovskite solar cells. Nature Photon 2014, 8, 506–514. 10.1038/nphoton.2014.134.

[ref45] Tan Z. K.; Moghaddam R.; Lai M.; et al. Bright light-emitting diodes based on organometal halide perovskite. Nat. Nanotechnol. 2014, 9, 687–692. 10.1038/nnano.2014.149. 25086602

[ref46] Stranks S.; Snaith H. Metal-halide perovskites for photovoltaic and light-emitting devices. Nat. Nanotechnol. 2015, 10, 391–402. 10.1038/nnano.2015.90. 25947963

[ref47] Wang N.; Cheng L.; Ge R.; et al. Perovskite light-emitting diodes based on solution-processed self-organized multiple quantum wells. Nat. Photon 2016, 10, 699–704. 10.1038/nphoton.2016.185.

[ref48] Dey A.; Ye J.; De A.; Debroye E.; Ha S. K.; Bladt E.; Kshirsagar A. S.; Wang Z.; Yin J.; Wang Y.; Quan L. N.; Yan F.; Gao M.; Li X.; Shamsi J.; Debnath T.; Cao M.; Scheel M. A.; Kumar S.; Polavarapu L. State of the Art and Prospects for Halide Perovskite Nanocrystals. ACS Nano 2021, 15 (7), 10775–10981. 10.1021/acsnano.0c08903. 34137264 PMC8482768

[ref49] Boehme S. C.; Bodnarchuk M. I.; Burian M.; Bertolotti F.; Cherniukh I.; Bernasconi C.; Zhu C.; Erni R.; Amenitsch H.; Naumenko D.; Andrusiv H.; Semkiv N.; John R. A.; Baldwin A.; Galkowski K.; Masciocchi N.; Stranks S. D.; Rainò G.; Guagliardi A.; Kovalenko M. V. Strongly Confined CsPbBr3 Quantum Dots as Quantum Emitters and Building Blocks for Rhombic Superlattices. ACS Nano 2023, 17 (3), 2089–2100. 10.1021/acsnano.2c07677. 36719353 PMC9933619

[ref50] Askes S. H. C.; Bahreman A.; Bonnet S. Activation of a Photodissociative Ruthenium Complex by Triplet–Triplet Annihilation Upconversion in Liposomes. Angew. Chem., Int. Ed. 2014, 53 (4), 1029–1033. 10.1002/anie.201309389. 24339049

[ref51] Cao X.; Hu B.; Zhang P. High Upconversion Efficiency from Hetero Triplet–Triplet Annihilation in Multiacceptor Systems. J. Phys. Chem. Lett. 2013, 4 (14), 2334–2338. 10.1021/jz401213w.

[ref52] La Rosa M.; Denisov S. A.; Jonusauskas G.; McClenaghan N. D.; Credi A. Designed Long-Lived Emission from CdSe Quantum Dots through Reversible Electronic Energy Transfer with a Surface-Bound Chromophore. Angew. Chem., Int. Ed. 2018, 57 (12), 3104–3107. 10.1002/anie.201712403. PMC5873259 29383800

[ref53] Montalti M.; Credi A.; Prodi L.; Gandolfi M. T. Handbook of Photochemistry, 3rd ed.; CRC Press, 2006 10.1201/9781420015195.

[ref54] Nishimura N.; Gray V.; Allardice J. R.; Zhang Z.; Pershin A.; Beljonne D.; Rao A. Photon Upconversion from Near-Infrared to Blue Light with TIPS-Anthracene as an Efficient Triplet–Triplet Annihilator. ACS Mater. Lett. 2019, 1 (6), 660–664. 10.1021/acsmaterialslett.9b00287.

[ref55] Mongin C.; Garakyaraghi S.; Razgoniaeva N.; Zamkov M.; Castellano F. N. Direct observation of triplet energy transfer from semiconductor nanocrystals. Science 2016, 351 (6271), 369–372. 10.1126/science.aad6378. 26798011

[ref56] Monguzzi A.; Mezyk J.; Scotognella F.; Tubino R.; Meinardi F. Upconversion-induced fluorescence in multicomponent systems: Steady-state excitation power threshold. Phys. Rev. B 2008, 78 (19), 195112 10.1103/PhysRevB.78.195112.

[ref57] Yurash B.; Dixon A.; Espinoza C.; Mikhailovsky A.; Chae S.; Nakanotani H.; Adachi C.; Nguyen T.-Q. Efficiency of Thermally Activated Delayed Fluorescence Sensitized Triplet Upconversion Doubled in Three-Component System. Adv. Mater. 2022, 34, 2103976 10.1002/adma.202103976. 34793602

[ref58] Protesescu L.; Yakunin S.; Bodnarchuk M. I.; Krieg F.; Caputo R.; Hendon C. H.; Yang R. X.; Walsh A.; Kovalenko M. V. Nanocrystals of Cesium Lead Halide Perovskites (CsPbX3, X = Cl, Br, and I): Novel Optoelectronic Materials Showing Bright Emission with Wide Color Gamut. Nano Lett. 2015, 15 (6), 3692–3696. 10.1021/nl5048779. 25633588 PMC4462997

[ref59] de Mello J. C.; Wittmann H. F.; Friend R. H. An improved experimental determination of external photoluminescence quantum efficiency. Adv. Mater. 1997, 9 (3), 230–232. 10.1002/adma.19970090308.

[ref60] Baluschev S.; Yakutkin V.; Miteva T.; Wegner G.; Roberts T.; Nelles G.; Yasuda A.; Chernov S.; Aleshchenkov S.; Cheprakov A. A general approach for non-coherently excited annihilation up-conversion: transforming the solar-spectrum. New J. Phys. 2008, 10 (1), 013007 10.1088/1367-2630/10/1/013007.

[ref61] Nienhaus L.; Correa-Baena J.-P.; Wieghold S.; Einzinger M.; Lin T.-A.; Shulenberger K. E.; Klein N. D.; Wu M.; Bulović V.; Buonassisi T.; Baldo M. A.; Bawendi M. G. Triplet-Sensitization by Lead Halide Perovskite Thin Films for Near-Infrared-to-Visible Upconversion. ACS Energy Lett. 2019, 4 (4), 888–895. 10.1021/acsenergylett.9b00283.

[ref62] Zhou Y.; Castellano F. N.; Schmidt T. W.; Hanson K. On the Quantum Yield of Photon Upconversion via Triplet–Triplet Annihilation. ACS Energy Lett. 2020, 5 (7), 2322–2326. 10.1021/acsenergylett.0c01150.

