# Supplementary material for: Multifold Enhanced Photon Upconversion in a Composite Annihilator System Sensitized by Perovskite Nanocrystals
Source: ACS Nano. 2024 May 31;18(23):15229–38. doi: 10.1021/acsnano.4c03753 (PMC11171765; doi:10.1021/acsnano.4c03753)
Supplement: Supplementary file 1 — nn4c03753_si_001.pdf [file nn4c03753_si_001.pdf]

Supporting Information

Multi-fold enhanced photon upconversion in a composite annihilator system sensitised by perovskite nanocrystals

Xian Wei Chua[+,1,2,3], *Linjie Dai*[+,1,3], *Miguel Anaya*[*,3,5], *Hayden Salway*[1,3], *Edoardo Ruggeri*[1], *Pengqing Bi*[2], *Zhihong Yang*[2], *Samuel D. Stranks*[*,1,3], *Le Yang*[*,2,4]

[1]Cavendish Laboratory, Department of Physics, University of Cambridge, JJ Thomson Avenue, Cambridge CB3 0HE, United Kingdom.

[2]Institute of Materials Research and Engineering (IMRE), Agency for Science, Technology and Research (A*STAR), Innovis #08-03, Singapore 138634, Singapore.

[3]Department of Chemical Engineering and Biotechnology, University of Cambridge, Philippa Fawcett Drive, Cambridge CB3 0AS, UK.

[4]Department of Materials Science and Engineering, National University of Singapore, 9 Engineering Drive 1, #03-09 EA, Singapore 117575, Singapore.

[5]Departamento Física de la Materia Condensada, Instituto de Ciencia de Materiales de Sevilla, Universidad de Sevilla−CSIC, Calle Américo Vespucio 49, Sevilla 41012, Spain.

[+]These authors contributed equally

*Corresponding authors: ma811@cam.ac.uk; sds65@cam.ac.uk; yang_le@imre.a-star.edu.sg

Section 1: Supplementary Figures

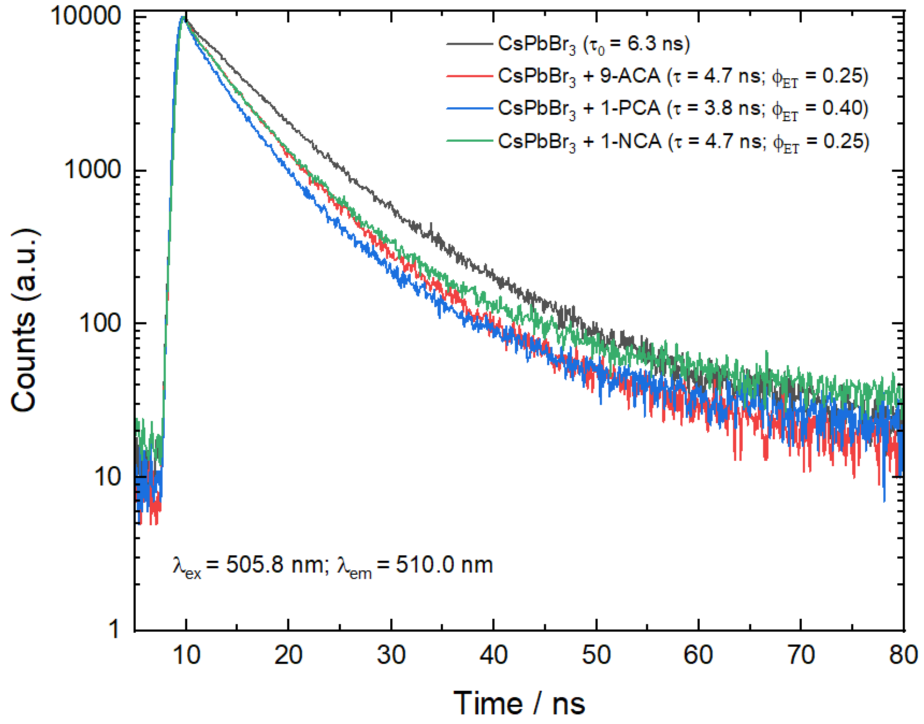

Figure S1. Time-correlated single photon counting (TCSPC) measurements to determine the choice of organic ligand. We measured the decay of the perovskite photoluminescence to gain insight into the energy transfer from the perovskite nanocrystals to organic ligands. Several carboxylate ligands were considered: 9-anthracenecarboxylicacid (9-ACA, red), 1-pyrenecarboxylicacid (1-PCA, blue), and 1-naphthalenecarboxylicacid (1-NCA, green). The energy transfer efficiency is estimated using $\Phi_{ET} = 1 - \tau/\tau_0$, where τ is the emission lifetime with the surface ligands, and τ_0 is the original emission lifetime. The optimisation experiments were carried out at the initial stages with $CsPbBr_3$ perovskite nanocrystals (5.6 mg/ml) and the organic ligands (0.95 mM) in anhydrous toluene. The laser excitation was 505.8 nm (repetition rate of 5 MHz at a fluence of 16.2 nJ/cm^2/pulse).

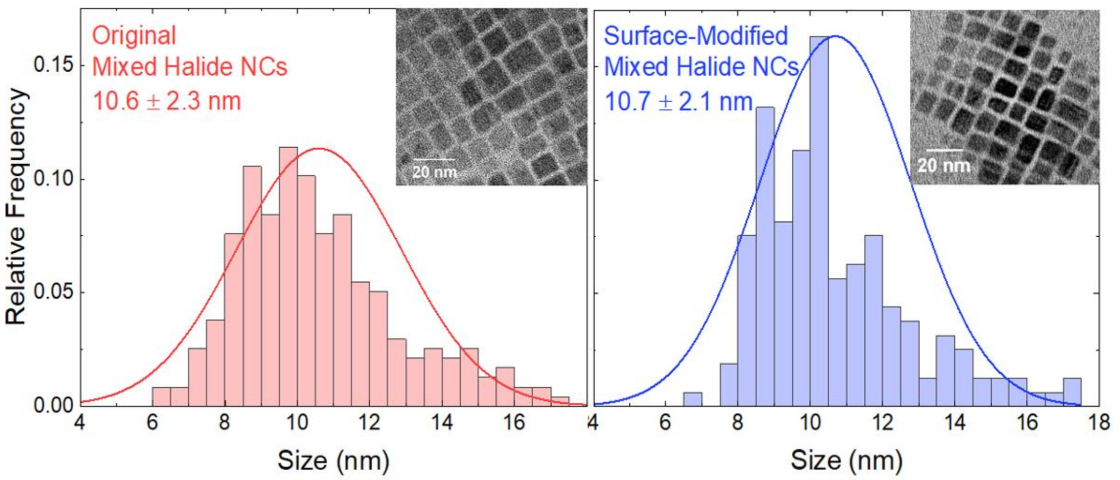

Figure S2. TEM measurement of original (left) and surface-modified (right) mixed halide nanocrystals (in toluene) used in the study. The size distribution statistics are each based on 80 nanocrystals measured laterally.

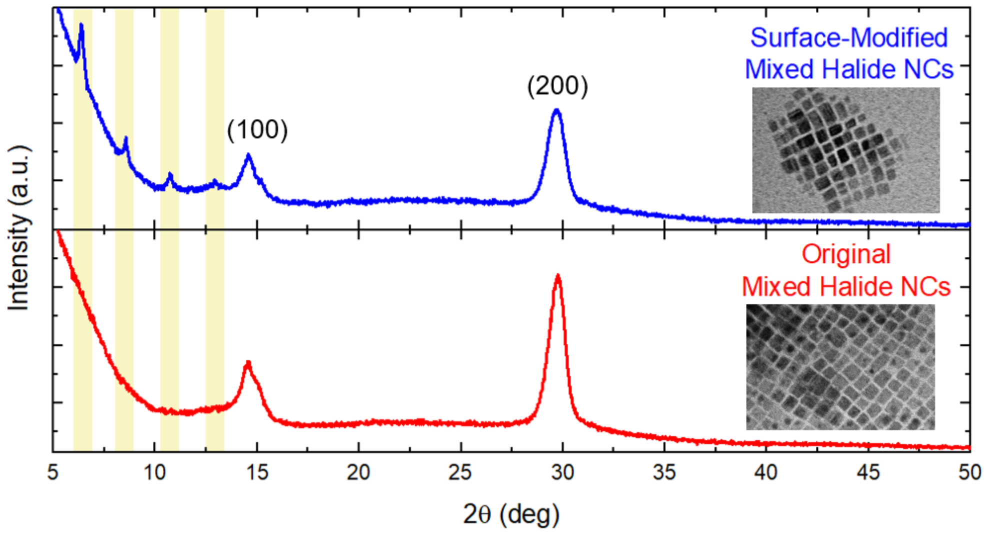

Figure S3. XRD patterns of surface-modified (top, blue line) and original (bottom, red line) mixed halide nanocrystals. There are additional low-dimensional X-ray diffraction components observed after surface modification with 1-PCA, indicated by the yellow shadings. The main (100) and (200) peaks at 14 and 29 2θ degrees are however preserved after surface treatment.

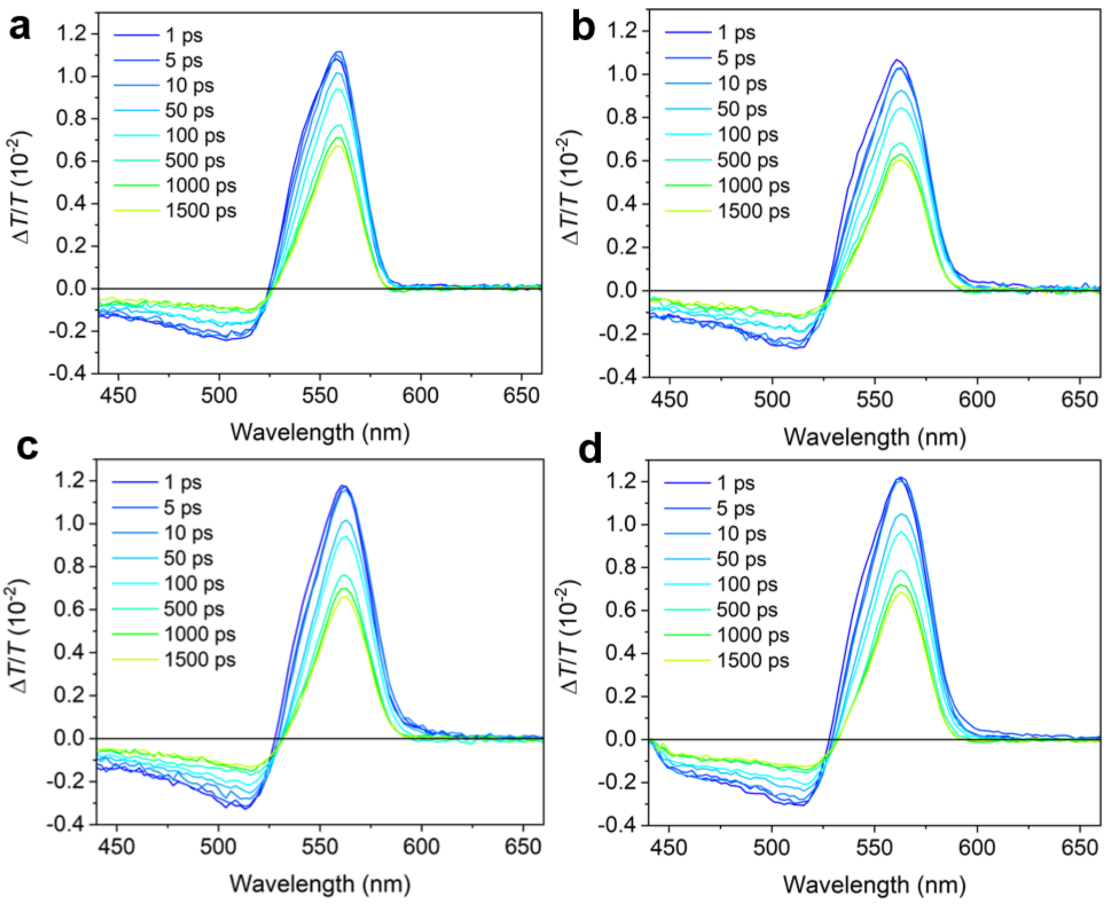

Figure S4. Transient absorption spectra (0 ps to 1850 ps) of (a) original and (b) surface-modified mixed halide nanocrystals (in toluene) under a 540 nm pump, with (c) a single acceptor of DPA (15 mM), or (d) a single acceptor of TIPS-An (1 mM).

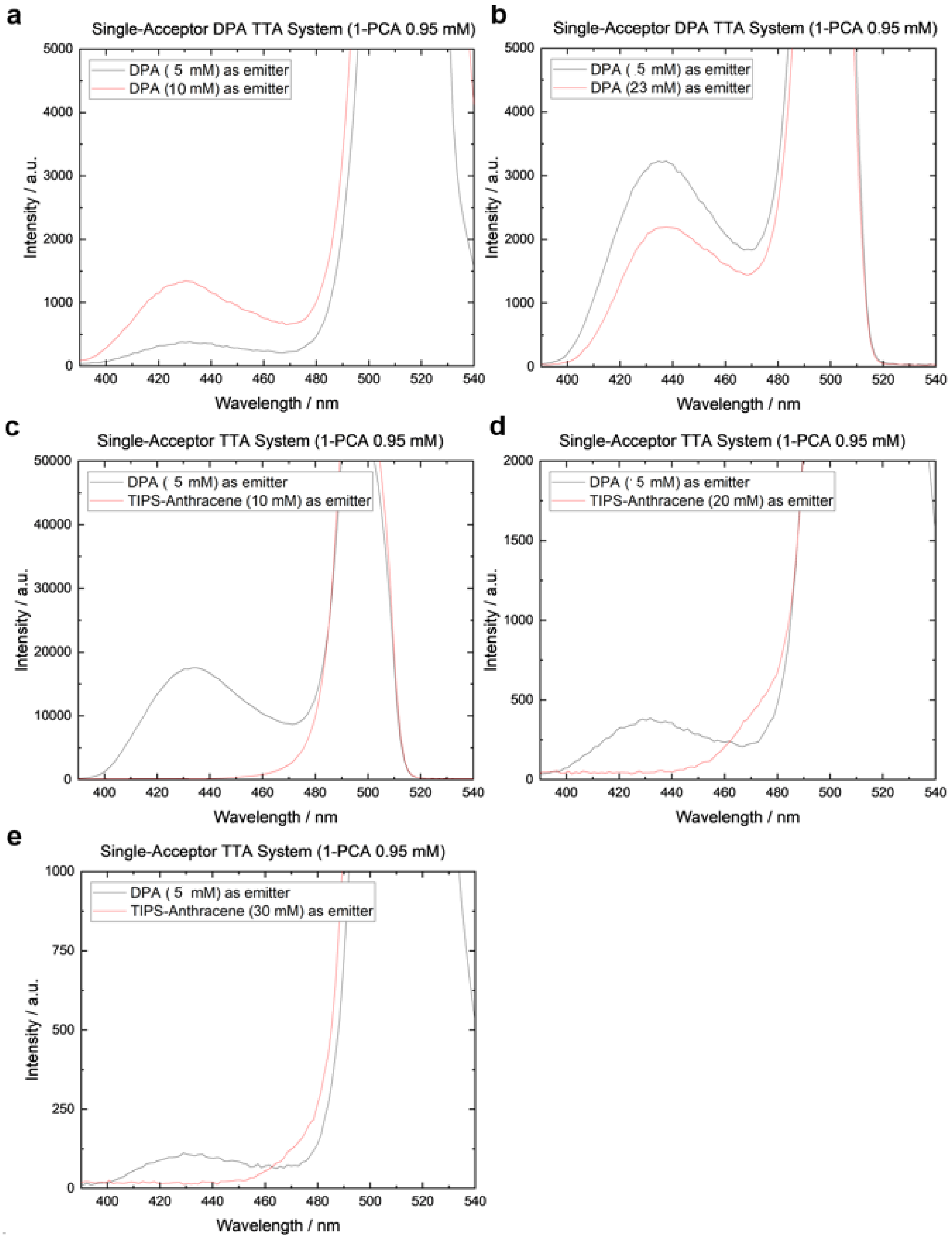

Figure S5. Preliminary optimisation data to obtain the concentrations of (a, b) DPA and (c, d, e) TIPS-An used in the single-acceptor TTA systems. All optimisation experiments were conducted using $CsPbBr_3$ (1.4 mg/ml) as the triplet sensitizer, and 1-PCA (0.95 mM) as the organic ligand. Single-acceptor DPA (5 mM) was used as the reference TTA system. A ThorLabs FES500 filter was used where necessary to avoid

the detector being saturated by the nanocrystals PL. The TTA signals of interest can be observed below 480 nm.

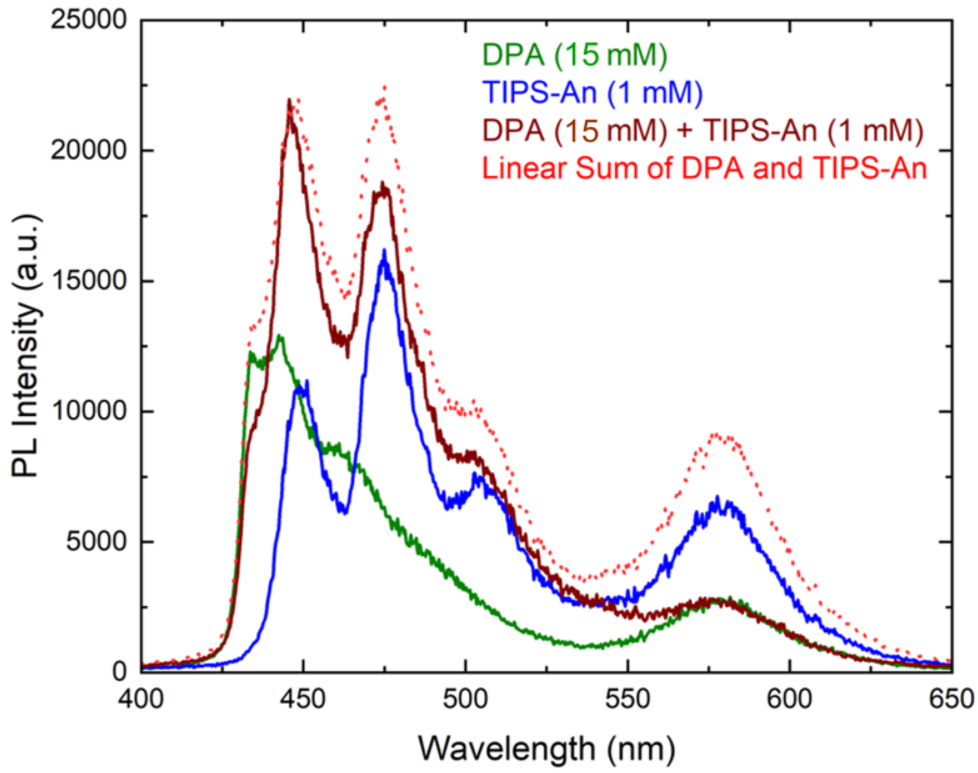

Figure S6. PL intensity using 400 nm pump for surface-modified mixed halide nanocrystals (in toluene) with single acceptor of DPA (15 mM) (green), single acceptor of TIPS-An (1 mM) (blue), and dual acceptors of DPA (15 mM) and TIPS-An (1 mM) (brown), collected using an iCCD camera. The red curve shows the linear sum of both single-acceptor systems.

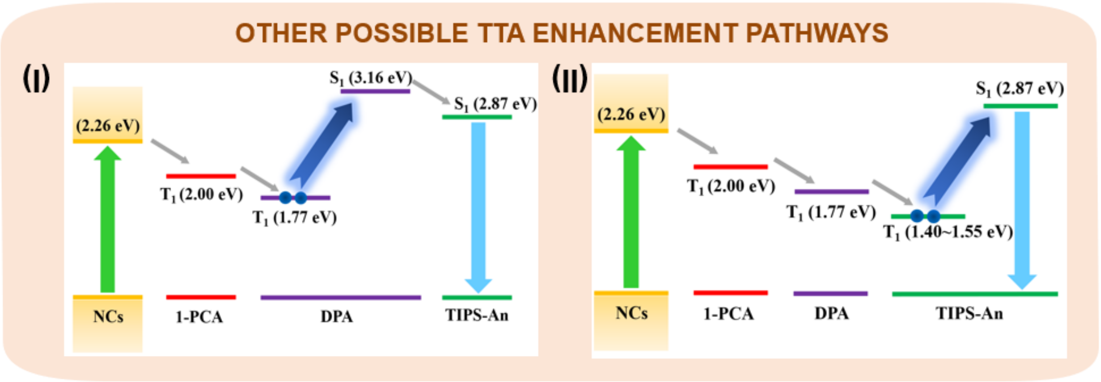

Figure S7. Possible *enhancement* mechanisms for observed enhanced TTA-UC in the composite system, although we believe these pathways do not play a significant role in accounting for the enhancement.

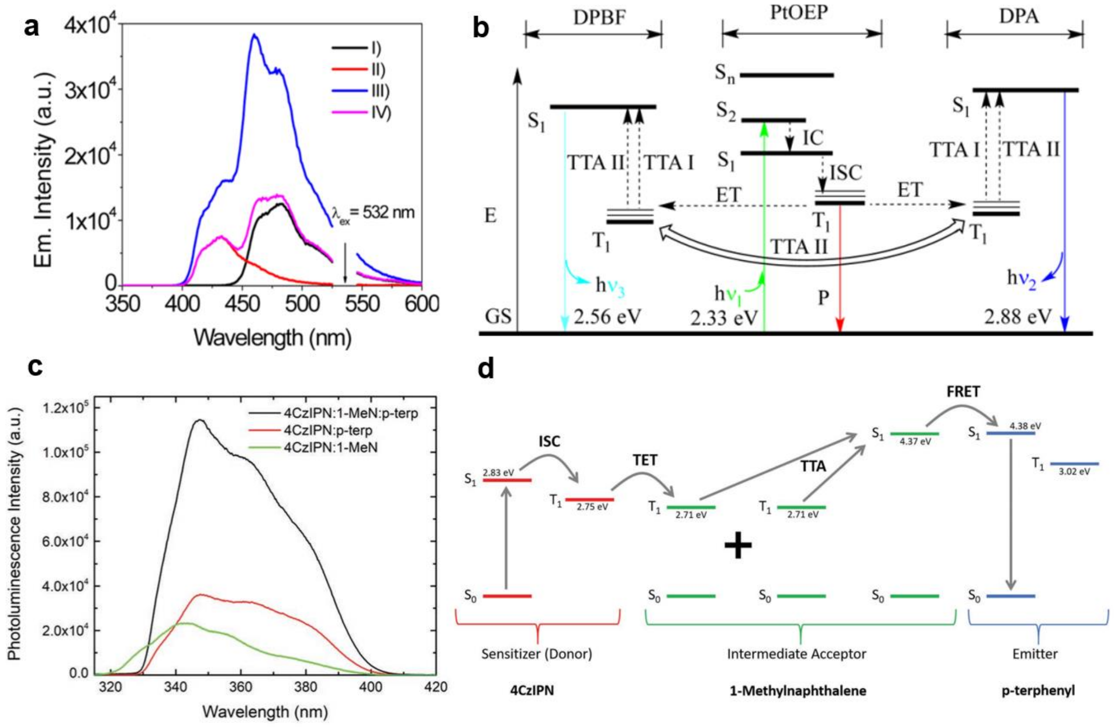

Figure S8. Comparison of our proposed mechanism and enhanced upconversion spectra with other related works [1-2]. (a) Enhanced upconversion spectra sensitised by a phosphor sensitiser, Platinum(II) octaethylporphyrin (PtOEP). The concentrations are

(I) DPBF (2 mM), (II) DPA (6 mM), (III) DPA (6 mM)/DPBF(2 mM), (IV) linear sum of (I) and (II). In (III), the upconversion emission from both DPA and DPBF are observed. The concentration of PtOEP is 25 μM in all samples. The solvent is N,N-dimethylformamide. (b) The proposed enhancement mechanism for panel (a), which is termed a “hetero-TTA” process between triplet acceptors of different types, indicated as “TTA II” on the panel. (a) and (b) are adapted with permission from [1]. Copyright 2013 American Chemical Society. (c) Enhanced upconversion spectra sensitised by a thermally activated delayed fluorescence sensitiser, 4,5,6-tetrakis(carbazol-9-yl)isophthalonitrile (4CzIPN). The concentrations are 25 mM p-terphenyl or 1-MeN in the binary solutions, and 25 mM 1-MeN and 26 mM p-terphenyl in the ternary solution where only the emission from p-terphenyl is observed. The concentration of 4CzIPN is 50 μM in all samples. The solvent is benzene. (d) The proposed enhancement mechanism for panel (c). (c) and (d) are adapted with permission from [2]. Copyright 2021 Wiley-VCH GmbH. Our work in fact combines features from both reports, applied to a perovskite-sensitised TTA-UC system, and achieves a five-fold enhancement compared to the sum of the individual systems. Our work, combined with earlier reports, demonstrates the general applicability of multi-acceptor systems in enhancing TTA-UC.

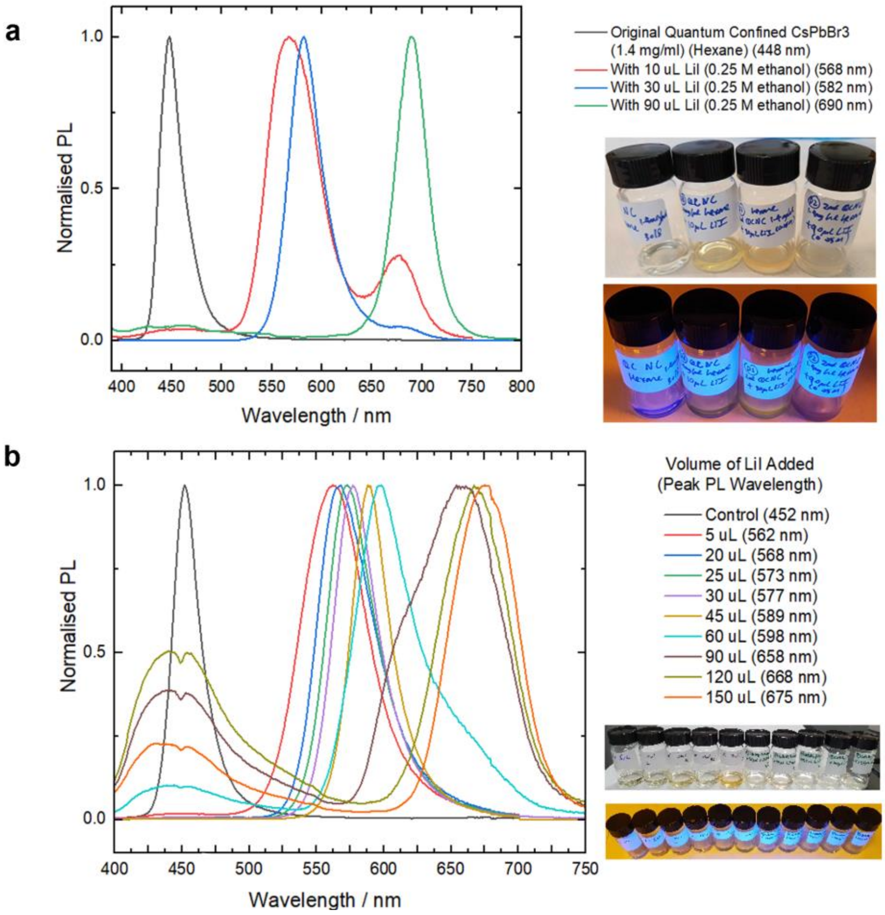

Figure S9. Halide exchange for 2 mL quantum-confined $CsPbBr_3$ nanocrystals (1.4 mg/mL in hexane), added with 5 to 150 µL LiI (0.25 M in ethanol), in an attempt to obtain quantum-confined mixed halide perovskite nanocrystals. The excitation was 380 nm. All the exchange reactions were carried out at room temperature in a N_2-filled glovebox. During an exchange reaction, the mixture was shaken vigorously, producing a color change towards red, with the peak PL wavelengths indicated in brackets. Two separate batches of nanocrystals – shown in (a) and (b) – both show the same outcome: that there is significantly reduced PLQE of the nanocrystals upon halide exchange, and there is persistently an unwanted broad PL signal at the original PL peak of the initial nanocrystals, suggesting incomplete halide exchange and chemical instability. These nanocrystals were not used subsequently for TTA-UC.

Section 2: Modelling the Composite Annihilator TTA-UC System

In a prototypical TTA-UC system involving an organometallic complex like platinum(II) octaethylporphyrin (PtOEP) as the triplet sensitizer and a single annihilator, the overall upconversion quantum yield is the product of the individual quantum yields of each step in the process [3-5]:

$$\phi_{UC,single} = f \cdot \phi_{ISC} \cdot \phi_{TET} \cdot \phi_{TTA} \cdot \phi_{f}$$

where ϕ_{ISC} is the intersystem crossing quantum yield of the sensitizer, ϕ_{TET} is the quantum yield of triplet energy transfer from sensitizer to annihilator, ϕ_{TTA} is the quantum yield of triplet-triplet annihilation, ϕ_{f} is the fluorescence quantum yield of the annihilator, and f is the spin statistical factor which describes the fraction of excited annihilator triplets that create an emissive singlet excited state following TTA. Since each TTA event consumes two triplet excited annihilators to produce one singlet excited annihilator (when f=1), the maximum value of ϕ_{TTA} and therefore ϕ_{UC} is 50%.

On the other hand, in our composite system of annihilators, we propose that the total upconversion quantum yield is now modified to the following equation:

$$\begin{aligned}\phi_{UC,composite} = {} & f_{DPA} \cdot \phi_{TET(NC\to 1-PCA)} \cdot \phi_{TET(1-PCA\to DPA)} \cdot \left[1 - \phi_{TET(DPA\to TIPS)}\right] \\ & \cdot \left[\phi_{TTA,DPA-DPA} + \phi_{TTA,composite}\right] \\ & \cdot \left[\left(1 - \phi_{FRET,DPA\to TIPS}\right) \cdot \phi_{f,DPA} + \phi_{FRET,DPA\to TIPS} \cdot \phi_{f,TIPS}\right] \\ & + f_{TIPS-An} \cdot \phi_{TET(NC\to 1-PCA)} \cdot \left[\phi_{TET(1-PCA\to TIPS-An)} + \phi_{TET(1-PCA\to DPA)} \right. \\ & \left. \cdot \phi_{TET(DPA\to TIPS)}\right] \cdot \left[\phi_{TTA,TIPS-TIPS} + \phi_{TTA,composite}\right] \cdot \phi_{f,TIPS}\end{aligned}$$

where the first term describes the case where S_1 (DPA) is first populated and the second term describes the case where S_1 (TIPS-An) is first populated following TTA.

$\phi_{FRET,DPA\rightarrow TIPS}$ describes the quantum yield of the ultrafast energy transfer from S_1 (DPA) to S_1 (TIPS-An) which we believe is close to unity, based on Figure 3a-c. $\phi_{TTA,DPA-DPA}$ and $\phi_{TTA,TIPS-TIPS}$ describe the quantum yield of triplet-triplet annihilation among DPA and TIPS-An molecules individually, respectively, while $\phi_{TTA,composite}$ represents the quantum yield of the composite triplet-triplet annihilation process between T_1 (DPA) and T_1 (TIPS-An). The combination of both terms in the equation now provide a pathway to achieving overall higher upconversion efficiencies.

Section 3: Additional Comments

1. In our manuscript, we use the terms "acceptor", "emitter", and "annihilator" interchangeably. DPA and TIPS-An play the role of triplet "acceptors" in the energetic design of our system, due to energy transfer from 1-PCA ligand. DPA and TIPS-An also behave as "emitters" when they emit the upconversion spectra measured, and as "annihilators" when participating in the mechanistic process of TTA.
2. We opted to synthesise $CsPbBr_{2.1}I_{0.9}$ nanocrystals, taking into account two considerations: to ensure PL energies do not overlap with the upconverted TTA signals, and to strike a balance between the size of the perovskite nanocrystals and their stability. For example, the exciton bleach feature at 496 nm and photo-induced absorption at 476 nm of the purely bromide nanocrystals nearly overlap

with the desired upconversion signals of the blue organic emitters DPA and TIPS-An, making the mixed-halide analogue more favourable.

3. The reabsorption by the triplet sensitizer is similar for all systems, whether single emitter DPA, single emitter TIPS-An, or both emitters, due to the same concentration of perovskite nanocrystals. In terms of the absorbance values plotted in Figure 1d, at 435 nm, the normalized absorbance is 0.15, while at 475 nm, the normalized absorbance is 0.10. This would not lead to the five times efficiency enhancement observed for the dual-acceptor system. Therefore, we do not believe absorption differences alone to be the cause for the observed enhancement.
4. The upconversion spectra in Figure 3d should be compared with the corresponding bottom-most spectra in Figures 3a-c with similar excitation densities. They adopt a similar spectral shape though we note that the spectra in Figure 3d appears less distinct. The reason is because low upconversion efficiencies require a long integration time and wide spectrometer slit settings.
5. In Figure 4, errors in the quantitative fitting of the slopes of the order ±0.02 to ±0.05 only propagate into small error margins in the reported threshold values.
6. We attempted time-resolved photoluminescence measurements employing an iCCD camera to elucidate the enhancement mechanism by exciting the samples with the highest fluence possible in the lab (up to 1 mJ/cm^2) using a Ti:Sapphire fundamental laser with a TOPAS amplifier system. However, due to the low UC efficiencies and short pulse width (<100 fs), we were not able to capture

direct time-resolved evidence for TTA. Upconversion is clearly observed under continuous-wave excitation such as seen in Figure 4.

7. We note that the photoluminescence quantum yield of DPA (ranging from 0.82 in benzene, to 0.95 in ethanol [6-8]) is higher than that of TIPS-Anthracene (0.75 in toluene [9]), and thus cannot account for the observed enhancement.
8. We note that the saturated Φ'_{UC} for our single-acceptor DPA system is lower than 1.3% obtained by Kimizuka and coworkers in a related system [10]. We suggest that their higher efficiency may perhaps be partly due to TTA between a surface-bound ligand resembling a DPA molecule and a free-floating DPA molecule [11], unlike between two free-floating DPA molecules in our case.
9. For the mixed halide perovskite nanocrystals as reference to calculate Φ'_{UC}, a smaller excitation wavelength of 520 nm was used instead of 532 nm, in order to minimise the emission peak of the nanocrystals overlapping with the laser peak. This allows us to separate the peaks and evaluate Φ'_{UC} more accurately. The different wavelengths are accounted for in the equation for Φ'_{UC} [12].
10. We acknowledge that Castellano *et al.* have utilized transient absorption spectroscopy to investigate the 1-PCA ligands [13-14]. They observed a band centred near 430 nm consistent with the $T_1 \rightarrow T_n$ transition of 1-PCA. However, the ^{3}DPA* characteristic $T_1 \rightarrow T_n$ transition absorption band also lies between 420-450 nm [15], consistent with another study on anthracene which found the triplet state exhibiting excited state absorption around 430 nm [16]. The congested bands may pose a challenge to differentiate the excited state

absorption among the various triplet acceptors 1-PCA, DPA and TIPS-An. Additionally, in our system, the low energy transfer efficiency from the perovskite nanocrystals to the 1-PCA ligands, and the low triplet concentrations, make it challenging for us to successfully observe the growth of the 1-PCA triplet band in the surface-modified mixed halide nanocrystals without emitter molecules, or the $T_1 \rightarrow T_n$ transition absorption band around 430 nm for the TTA systems, on our nanosecond to microsecond TA spectroscopy.

11. While Castellano *et al.* observed Dexter-like triplet-triplet energy transfer (TTET) from the nanocrystals to surface-bound acceptor molecules with near unity efficiency [13-14], there are in general various factors that affect the efficiency of the TTET process [17]. In particular, as stated in the main text, for efficient triplet energy transfer from the perovskite nanocrystals to polycyclic aromatic hydrocarbons, Wu *et al.* have shown that quantum confinement is required [18-20]. This could be a reason for the low energy transfer efficiency of 12% obtained in the main text. We were not able to synthesize quantum-confined yet stable perovskite nanocrystals which emit in the wavelength desired around 568 nm. While small nanocrystals (such as 3.5 nm) can facilitate efficient triplet energy transfer, their PL peak (such as 454 nm, corresponding to 3.5 nm size) [18] would overlap with the upconverted signals of the blue TTA emitters used in this work. We attempted a halide exchange procedure [21] to quantum-confined perovskite nanocyrstals at our desired photoluminescence wavelength, however they showed a significantly reduced PLQE upon halide

exchange and chemical instability (Figure S9), and were subsequently not used for TTA-UC.

12. We note that our absorption and photoluminescence spectra of pristine 1-PCA solution presented in Figure 1a are consistent with [22] and [23]. The 1-PCA absorption peaks at 356 nm, 366 nm and 386 nm do not overlap with the broad photoluminescence of the mixed halide perovskite nanocrystals centred at 567 nm. Singlet energy transfer from the nanocrystals to the 1-PCA ligands is not thermodynamically favourable.
13. As stated in the main text, in TTA-UC systems, we expect a quadratic to linear transition in the TTA-UC emission intensity with excitation power, attributed to monomolecular triplet decays and bimolecular TTA at low and high excitation intensities, respectively. The slopes we obtained in Figure 4 may deviate due to other higher-order processes, such as singlet-singlet annihilation or triplet-charge annihilation [24], although the exact reasons are unclear and requires investigation beyond the scope of this work.

References

1. Cao, X., Hu, B., & Zhang, P. (2013). High Upconversion Efficiency from Hetero Triplet–Triplet Annihilation in Multiacceptor Systems. *The Journal of Physical Chemistry Letters*, *4*(14), 2334-2338. https://doi.org/10.1021/jz401213w

2. Yurash, B., Dixon, A., Espinoza, C., Mikhailovsky, A., Chae, S., Nakanotani, H., Adachi, C., Nguyen, T.-Q., Efficiency of Thermally Activated Delayed Fluorescence Sensitized Triplet Upconversion Doubled in Three-Component System. *Adv. Mater.* 2022, 34, 2103976. https://doi.org/10.1002/adma.202103976
3. Edhborg, F., Olesund, A. & Albinsson, B. Best practice in determining key photophysical parameters in triplet–triplet annihilation photon upconversion. Photochem Photobiol Sci 21, 1143–1158 (2022). https://doi.org/10.1007/s43630-022-00219-x
4. Olesund, A., Gray, V., Mårtensson, J., & Albinsson, B. (2021). Diphenylanthracene Dimers for Triplet–Triplet Annihilation Photon Upconversion: Mechanistic Insights for Intramolecular Pathways and the Importance of Molecular Geometry. Journal of the American Chemical Society, 143(15), 5745–5754. https://doi.org/10.1021/jacs.1c00331
5. Turshatov, A., Busko, D., Avlasevich, Y., Miteva, T., Landfester, K. and Baluschev, S. (2012), Synergetic Effect in Triplet–Triplet Annihilation Upconversion: Highly Efficient Multi-Chromophore Emitter. ChemPhysChem, 13: 3112-3115. https://doi.org/10.1002/cphc.201200306
6. Morris, J. V., Mahaney, M. A., & Huber, J. R. (1976). Fluorescence quantum yield determinations. 9,10-Diphenylanthracene as a reference standard in different solvents. *The Journal of Physical Chemistry, 80*(9), 969-974. https://doi.org/10.1021/j100550a010

7. Heinrich, G., Schoof, S., & Gusten, H. (1974). 9,10-diphenylanthracene as a fluorescence quantum yield standard. *Journal of Photochemistry*, *3*(2), 315-320. https://doi.org/https://doi.org/10.1016/0047-2670(74)80040-7
8. Brouwer, A. (2011). Standards for photoluminescence quantum yield measurements in solution (IUPAC Technical Report). *Pure and Applied Chemistry*, *83*(12), 2213-2228. https://doi.org/10.1351/PAC-REP-10-09-31
9. Nishimura, N., Gray, V., Allardice, J. R., Zhang, Z., Pershin, A., Beljonne, D., & Rao, A. (2019). Photon Upconversion from Near-Infrared to Blue Light with TIPS-Anthracene as an Efficient Triplet–Triplet Annihilator. *ACS Materials Letters*, *1*(6), 660-664. https://doi.org/10.1021/acsmaterialslett.9b00287
10. Mase, K., Okumura, K., Yanai, N., & Kimizuka, N. (2017). Triplet sensitization by perovskite nanocrystals for photon upconversion. *Chemical Communications*, *53*(59), 8261-8264. https://doi.org/10.1039/C7CC03087H
11. Ni, L. (2020). Exciton and Energy Transfer Dynamics in Hybrid Perovskite and Lanthanide Nanomaterials. https://doi.org/10.17863/CAM.48588
12. Baluschev, S., Yakutkin, V., Miteva, T., Wegner, G., Roberts, T., Nelles, G., Yasuda, A., Chernov, S., Aleshchenkov, S., & Cheprakov, A. (2008). A general approach for non-coherently excited annihilation up-conversion: transforming the solar-spectrum. *New Journal of Physics*, *10*(1), 013007. https://doi.org/10.1088/1367-2630/10/1/013007

13. Mongin, C., Garakyaraghi, S., Razgoniaeva, N., Zamkov, M., & Castellano, F. N. (2016). Direct observation of triplet energy transfer from semiconductor nanocrystals. Science, 351(6271), 369-372. https://doi.org/doi:10.1126/science.aad6378
14. Mongin, C., Moroz, P., Zamkov, M., & Castellano, F. N. (2018). Thermally activated delayed photoluminescence from pyrenyl-functionalized CdSe quantum dots. *Nature Chemistry*, *10*(2), 225-230. https://doi.org/10.1038/nchem.2906
15. Yang, M., Sheykhi, S., Zhang, Y., Milsmann, C., & Castellano, F. N. (2021). Low power threshold photochemical upconversion using a zirconium(iv) LMCT photosensitizer. *Chemical Science, 12*(26), 9069–9077. https://doi.org/10.1039/D1SC01662H
16. Lang, B., Mosquera-Vázquez, S., Lovy, D., Sherin, P., Markovic, V., & Vauthey, E. (2013). Broadband ultraviolet-visible transient absorption spectroscopy in the nanosecond to microsecond time domain with sub-nanosecond time resolution. *Review of Scientific Instruments*, *84*(7), 073107. https://doi.org/10.1063/1.4812705
17. Dexter, D. L. (2004). A Theory of Sensitized Luminescence in Solids. The Journal of Chemical Physics, 21(5), 836–850. https://doi.org/10.1063/1.1699044
18. Luo, X., Lai, R., Li, Y., Han, Y., Liang, G., Liu, X., Ding, T., Wang, J., & Wu, K. (2019). Triplet Energy Transfer from CsPbBr3 Nanocrystals Enabled by

Quantum Confinement. *Journal of the American Chemical Society*, *141*(10), 4186-4190. https://doi.org/10.1021/jacs.8b13180

19. He, S., Luo, X., Liu, X., Li, Y., & Wu, K. (2019). Visible-to-Ultraviolet Upconversion Efficiency above 10% Sensitized by Quantum-Confined Perovskite Nanocrystals. *The Journal of Physical Chemistry Letters*, *10*(17), 5036-5040. https://doi.org/10.1021/acs.jpclett.9b02106
20. Han, Y., Luo, X., Lai, R., Li, Y., Liang, G., & Wu, K. (2019). Visible-Light-Driven Sensitization of Naphthalene Triplets Using Quantum-Confined CsPbBr3 Nanocrystals. The Journal of Physical Chemistry Letters, 10(7), 1457–1463. https://doi.org/10.1021/acs.jpclett.9b00597
21. Ramasamy, P., Lim, D.-H., Kim, B., Lee, S.-H., Lee, M.-S., & Lee, J.-S. (2016). All-inorganic cesium lead halide perovskite nanocrystals for photodetector applications. *Chem. Commun.*, *52*(10), 2067–2070. https://doi.org/10.1039/C5CC08643D
22. Krawczyk, S., Nawrocka, A., & Zdyb, A. (2018). Charge-transfer excited state in pyrene-1-carboxylic acids adsorbed on titanium dioxide nanoparticles. *Spectrochimica Acta Part A: Molecular and Biomolecular Spectroscopy*, *198*, 19–26. https://doi.org/10.1016/j.saa.2018.02.061
23. Zelent, B., Vanderkooi, J. M., Coleman, R. G., Gryczynski, I., & Gryczynski, Z. (2006). Protonation of Excited State Pyrene-1-Carboxylate by Phosphate and Organic Acids in Aqueous Solution Studied by Fluorescence

Spectroscopy. *Biophysical Journal*, *91*(10), 3864–3871. https://doi.org/10.1529/biophysj.106.088740

24. Izawa, S., Hiramoto, M. Efficient solid-state photon upconversion enabled by triplet formation at an organic semiconductor interface. Nat. Photon. 15, 895–900 (2021). https://doi.org/10.1038/s41566-021-00904-
